# Multi‐Tissue Omics Analysis Uncovers Translational Regulation Underlying Complex Traits in Pigs

**DOI:** 10.1002/advs.74200

**Published:** 2026-02-03

**Authors:** Chao Wang, Yuanyuan Zhang, Choulin Chen, Xiaoxian Xu, Shenghua Qin, Junyan Qi, Yongzhou Bao, Huiming Li, Ruipu Chen, Weigang Zheng, Zhilong Chen, Lingzhao Fang, Yunxiang Zhao, Yuwen Liu

**Affiliations:** ^1^ State Key Laboratory of Genome and Multi‐omics Technologies Shenzhen Branch Guangdong Laboratory for Lingnan Modern Agriculture Key Laboratory of Livestock and Poultry Multi‐Omics of MARA Agricultural Genomics Institute at Shenzhen Chinese Academy of Agricultural Sciences Shenzhen China; ^2^ Innovation Group of Pig Genome Design and Breeding Research Centre for Animal Genome Agricultural Genomics Institute at Shenzhen Chinese Academy of Agricultural Sciences Shenzhen China; ^3^ Guangxi Key Laboratory of Animal Breeding Disease Control and Prevention College of Animal Science and Technology Guangxi University Nanning China; ^4^ Center For Quantitative Genetics and Genomics (QGG) Aarhus University Aarhus Denmark

**Keywords:** gene regulatory network, pig, post‐transcriptional regulation, translation, variants

## Abstract

Variations in both transcription and translation of genes play a pivotal role in shaping complex traits and disease phenotypes. However, systematic analyses of genetic variants regulating transcription and translation, as well as their contribution to the genetic architecture of complex traits, remain scarce. Here, by generating a multi‐omics dataset consisting of 132 datasets (48 transcriptomic, 48 translational, 30 proteomic, and 6 WGS) across 16 tissues from two breeds, with 3 pigs per breed, we demonstrated widespread translational buffering/amplification across tissues and breeds, with translation efficiency (TE) contributing significantly to phenotypic variation. Through integrative analysis of transcriptional and translational profiles, population genetics, and dual‐luciferase reporter assays, we developed a novel framework for prioritizing gene regulatory networks (GRNs) underlying complex traits. Using this framework, we identified 33 functional 5’UTR variants linked to pork production traits, modulating 14 target genes through changes in TE. RNA interference assays confirmed the involvement of *AQP4* and *MYO18B* in myogenic differentiation. In particular, the *AQP4* variant (chr6_111421187) likely alters TE by modifying RNA secondary structure, while *MYO18B* variants (chr14_43476491) affect TE via RNA‐binding protein interactions.

More broadly, our framework can serve as a paradigm for uncovering the genetic basis of complex traits, extending beyond traditional transcriptional regulation.

## Graphical abstract

Integrated multi‐omics across pig breeds and tissues reveals key principles of post‐transcriptional translational regulation and their contribution to trait formation. A gene network framework integrating transcriptomic and translatomic data is developed and applied to elucidate the genetic basis of meat production, offering new insights into mammalian trait architecture.

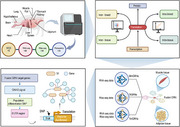

## 1 Introduction

Precise regulation of protein abundance at multiple levels is fundamental to the manifestation of complex traits. Although protein levels are shaped by a cascade of regulatory processes—including transcription, splicing, mRNA degradation, translation, post‐translational modifications, and protein degradation—research has predominantly focused on mechanisms controlling RNA abundance. In various species, RNA sequencing (RNA‐seq) [1] has facilitated the construction of comprehensive gene expression atlases, offering valuable insights into the transcriptional regulatory networks underlying complex phenotypes [2, 3, 4, 5, 6]. To further uncover the genetic basis of mRNA abundance, population‐scale expression quantitative trait loci (eQTL) studies in multiple species—such as those from the Genotype‐Tissue Expression (GTEx) project [7] and FarmGTEx [8, 9, 10, 11]—have systematically identified genetic variants associated with gene expression levels, capturing the combined influences of transcription, splicing, and mRNA stability. These efforts have significantly advanced our understanding of how genetic variants influence complex traits through regulating the transcriptional process. However, despite this progress, far less attention has been given to how genetic variants modulate phenotypic variation through translational regulation, leaving a critical gap in our understanding of the full regulatory architecture of complex traits.

Recent studies across various species show that within specific tissues or samples, the correlation between mRNA and protein levels typically ranges from 0.4 to 0.8 [12, 13, 14, 15, 16, 17, 18, 19, 20]. However, at the population level, within‐gene correlations between mRNA and protein abundance are generally weaker, with median correlation coefficients ranging from 0.14 to 0.59 [18, 20, 21, 22, 23, 24, 25, 26, 27, 28, 29]. These observations highlight the discordance between mRNA and protein levels, emphasizing the crucial role of post‐transcriptional regulation in shaping protein abundance. This discrepancy raises an important question: how well do inherited eQTLs correlate with proteomic variation, and to what extent do translational regulations contribute? Notably, studies have shown that eQTLs exhibit limited overlap with protein quantitative trait loci (pQTLs) and loci of complex traits [30]. For instance, the overlap is reported to be 35% in human lymphoblastoid cell lines [21], 32% in mouse liver [31], 5.5% in inbred and recombinant mice [32], and just 3.6% in yeast [20]. Moreover, a recent study mapping eQTLs, rQTLs (ribosome‐occupancy QTLs), and pQTLs in the human prefrontal cortex demonstrated a progressive decline in the number of significant QTLs when moving downstream along the central dogma, revealing substantial translational and post‐translational attenuation of eQTL effects [33]. Collectively, these findings suggest that traditional eQTL approaches do not fully capture the variation in protein abundance, underscoring the importance of identifying genetic variants that influence protein levels independently of mRNA abundance. In this context, previous studies have identified variants within the 5’UTR that affect TE [34, 35]. However, systematic investigations into such variants remain limited, especially when compared to the extensive research focused on those affecting mRNA abundance. This gap hinders our understanding of how transcription and post‐transcriptional regulation are coordinated to control fundamental biological processes and complex traits.

To construct a translational regulatory landscape, Ribo‐seq, a high‐throughput technique that deep‐sequence ribosome‐protected fragments (RPFs) [36], has been increasingly applied in various tissues across species, such as mouse [37], human [38], maize [39], Arabidopsis thaliana [40], bread wheat [41], and rice [42]. This technique enables comprehensive in vivo monitoring of translational processes and can thus serve as a proxy for protein synthesis [43]. When integrated with RNA sequencing, Ribo‐seq facilitates genome‐wide quantification of TE, typically represented by the ratio of RPFs to RNA abundance [36]. While RNA levels reflect transcriptional activity, RPFs capture translational output, together revealing widespread post‐transcriptional regulation that often explains the discordance between mRNA and protein levels [44]. Although these integrative approaches have significantly advanced our understanding of translational dynamics across biological contexts, most studies have been limited to descriptive comparisons of transcriptional and translational profiles across tissues or under stress conditions. Two key challenges remain: first, the limited use of proteomic data to validate how TE differences contribute to protein abundance variation [45]; second, the lack of systematic investigation into how genetic variation influences complex traits through translational regulation, leaving the genetic architecture of translation largely unexplored in polygenic trait contexts.

Pigs encompass a wide range of breeds with pronounced phenotypic differences between Eastern and Western varieties [46, 47, 48], making them an excellent model for studying the interplay between translational control and mRNA abundance in the regulation of complex traits. For instance, Eastern breeds are typically characterized by superior meat quality and increased fat deposition, whereas Western breeds are known for their rapid growth rates and high lean meat yield [47, 48]. Comprehensive transcriptional [49] and epigenomic [50] maps across multiple pig tissues and breeds have illuminated potential causal variants influencing mRNA levels, thereby laying a strong foundation for exploring additional regulatory layers at the translational level. Moreover, due to their physiological and anatomical similarities to humans, pigs serve as valuable models for biomedical research. This is exemplified by their use in studies of human diseases such as Alzheimer's disease [51, 52], Muscular dystrophy [53], Diabetes mellitus [54], cardiovascular diseases [55], and obesity [56]. Despite these advantages, the multi‐tissue translational landscape in pigs remains largely uncharted, posing a major gap in leveraging this model organism to investigate the role of translational regulation in complex trait formation.

In this study, we generated comprehensive whole‐transcriptome RNA‐seq and Ribo‐seq profiles from 16 tissues of two pig breeds: Ba Ma (BM), a small‐bodied indigenous breed from southern China, and Large White (LW), a lean commercial breed. These breeds represent Eastern and Western pig lineages, respectively, providing a unique opportunity to explore gene transcriptional and translational expression diversity across tissues and genetic backgrounds, as well as the contribution of post‐transcriptional regulation to phenotypic variation. By integrating transcriptional and translational profiling with population genetics and validating the translational effects of 5’UTR variants through functional assays, we refined the construction of GRNs to incorporate translational regulation. This study not only provides a theoretical framework for the genetic improvement of pork production traits but also introduces a novel paradigm for exploring the genetic architecture of complex traits through the lens of translational regulation.

## 2 Results

### 2.1 Atlas of Transcriptome, Translatome, and Proteome Profiling Across 16 Pig Tissues

**FIGURE 1:**
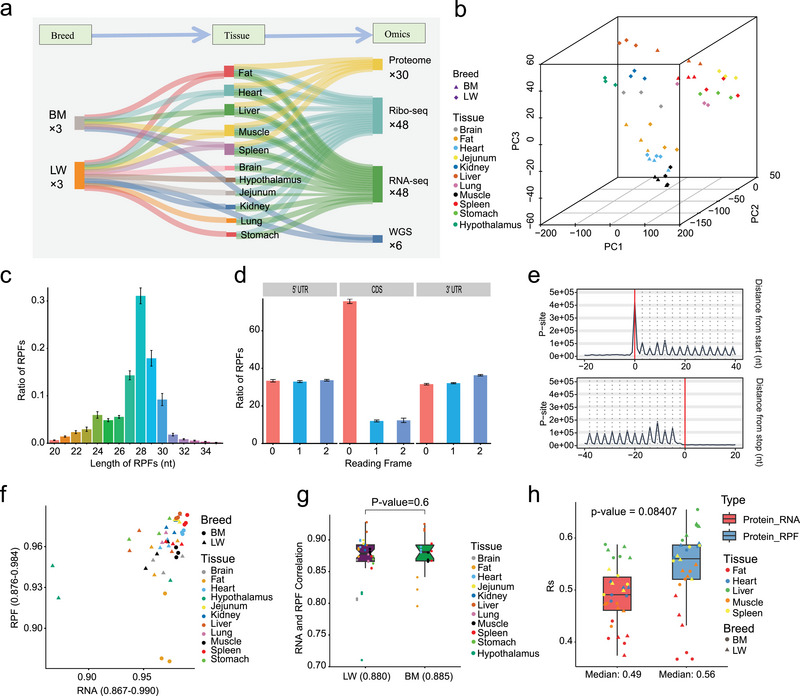
Multi‐omics dataset characterization and translatome features. (a) A Sankey diagram illustrating the overall information of the samples used and multi‐omics sequencing data. (b) 3D PCA plot illustrating the biological replicability of the translatome data. (c) Length distribution of RPFs, with a prominent peak at 28 nt. Error bars represent the mean ± standard error (SE). (d) Ratio of RPFs in the 0, 1, and 2 reading frames across the 5’UTR, CDS, and 3’UTR regions. Error bars represent the mean ± SE. (e) Distribution of optimally mapped reads along the CDS, with each read represented by its P‐site position based on fragment length. (f) Scatter plot illustrating the correlation between biological replicates within the same tissue at the RNA and RPF levels. The *x*‐axis represents the correlation values for RNA, while the *y*‐axis represents the correlation values for RPFs. The biological replicate correlation for RNA ranges from 0.867 to 0.990, and for RPFs, it ranges from 0.867 to 0.984. Correlations were obtained using the Pearson correlation test. (g) Comparison of Pearson correlation coefficients (r) between RNA and RPF within samples from different tissues across breeds. *p*‐values were calculated using the Mann–Whitney U test. (h) Comparison of r between RNA vs. protein and RPF vs. protein across different tissue samples. The median Pearson correlation coefficient for RNA vs. protein was 0.49, while for RPF vs. protein it was 0.56. *p*‐values were calculated using the Mann–Whitney U test.

To systematically characterize gene translational dynamics across tissues and breeds and to elucidate their post‐transcriptional contributions to phenotypic variation, we generated comprehensive multi‐omics datasets from LW and BM pigs. These datasets include transcriptomic, translatomic, proteomic, and WGS data. LW pigs were sampled for 11 tissues, and BM pigs for 5 tissues, each with three biological replicates (Figure 1a). Ribo‐seq produced over 5.39 billion raw reads (∼112.4 million per library; Table S1), and RNA‐seq yielded over 2.1 billion reads (∼43.8 million per library; Table S2). After quality control (Methods), clustering analyses at the translatome (Figure 1b), transcriptome (Figure S1a), and proteome (Figure S1b) levels demonstrated high concordance among replicates. While RPFs in mammals typically range from 28 to 30 nucleotides (nt) [37, 57], in pigs, we observed a dominant RPF size of 27 to 30 nt (Figure 1c), which displayed clear 3‐nt periodicity (Figure 1e; Figure S2) and predominantly mapped to the primary reading frames within coding sequences (CDSs) (Figure 1d). High reproducibility across biological replicates was confirmed by strong Pearson correlation coefficients (0.88–0.98 for Ribo‐seq and 0.87–0.99 for RNA‐seq), with most tissues exhibiting r > 0.9 (Figure 1f). Together, these results confirm the generation of high‐quality, reproducible multi‐omics data suitable for downstream analyses.

We next examined correlations between RPF and mRNA abundances within individual samples. In most tissues, these within‐sample correlations were below 0.9 (LW: median r = 0.880; BM: median r = 0.885), with no significant difference between the two breeds (*p* = 0.6), indicating post‐transcriptional regulation contributes to discrepancies between transcriptional and translational outputs (Figure 1g). We further assessed the relationship between RPF, mRNA, and protein abundances both within and across tissues. Notably, cross‐tissue RPF–protein correlations were significantly higher than mRNA–protein correlations (*p* = 8.5 × 10^−^^6^) (Figure S3). Although the within‐tissue differences between RPF–protein and mRNA–protein correlations did not reach statistical significance (*p* = 0.08), RPF–protein correlations were generally stronger across most tissues, except for adipose tissue in BM pigs (Figure 1h). These results suggest that RPF levels serve as a more accurate proxy for protein abundance than mRNA levels, offering a valuable perspective for investigating the translational mechanisms underlying complex trait formation.

### 2.2 Translation Regulation as a Mechanism for Fine‐Tuning Transcriptional Signals

**FIGURE 2:**
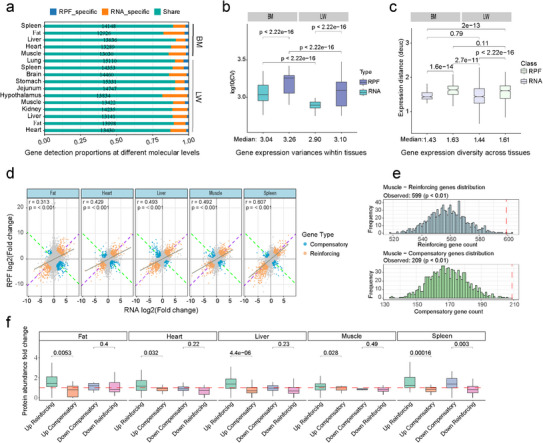
Gene expression diversity at mRNA and RPF levels across tissues and breeds. (a) Number of genes detected in different tissues by RNA‐seq and Ribo‐seq. Blue represents genes detected exclusively by RPF, orange represents genes detected only by mRNA, and green represents genes detected by both RPF and mRNA. (b) Comparison of gene expression variability within tissues at the mRNA and RPF levels. CV values for both RNA and RPF were calculated across 17, 206 genes (the union of genes with detectable expression in any tissue) within each tissue. Boxplots show the 25th percentile, median, and 75th percentile. *p*‐values were calculated using the Mann–Whitney U test. (c) Boxplots showing the Euclidean expression distance between different tissues within the same breed at both RNA and RPF levels. The *x*‐axis indicates the median Euclidean distance across all tissue pairs within each breed. Differences between groups were evaluated using the Wilcoxon rank‐sum test. (d) Scatterplots of mRNA log_2_FC versus RPF log_2_FC for genes in five tissues (LW vs. BM). Orange points indicate genes exhibiting reinforcing interactions (two‐standard‐deviation distance from the origin along the purple dashed line, *y* = *x*), blue points indicate genes with compensatory interactions (two‐standard‐deviation distance from the origin along the green dashed line, *y* = −*x*), and gray points indicate genes that did not meet the significance thresholds for interaction. The r in the top‐left corner represents the PCC between mRNA log2FC and RPF log2FC across all genes in this tissue. (e) Frequency distributions showing the number of reinforcing (top panel) and compensatory (bottom panel) genes across 1000 randomized datasets; red dashed lines indicate the observed counts in the empirical data, with significant enrichment defined as *p* < 0.01. (f) Boxplots showing protein FC between breeds for genes classified as reinforcing or compensatory. The direction of change (Up or Down) is defined based on the mRNA log_2_FC (LW vs. BM). Differences between groups were evaluated using a two‐sided *t*‐test.

To systematically investigate gene expression variation at both transcriptional and translational levels—within and across tissues, as well as between breeds—we independently quantified mRNA and RPF abundance for each tissue. Overall, more genes were detected at the mRNA level in each tissue (Figure 2a). To assess the contribution of translation to gene expression variability, we calculated the coefficient of variation (CV) for mRNA and RPF levels within each tissue. In both breeds, RPF CVs were consistently higher than mRNA CVs (Figure 2b; *p* < 2.22e−16), highlighting the substantial role of translational regulation. We next assessed cross‐tissue transcriptional and translational divergence by calculating pairwise Euclidean distances between tissues within each breed (Figure 2c). In both breeds, divergence at the mRNA level was significantly lower than at the RPF level, with the median RNA‐based divergence reaching 88% of the RPF‐based divergence in BM pigs and 89% in LW pigs. This suggests the involvement of post‐transcriptional mechanisms, particularly translational regulation, in buffering or amplifying transcriptional signals. Notably, there were no significant breed‐specific differences in divergence at either mRNA or RPF levels, indicating that cross‐tissue divergence is conserved across breeds. We further examined divergence in gene expression across breeds within the same tissue type (Figure S4). Both mRNA (median: 0.68) and RPF (median: 0.77) divergences were significantly lower than cross‐tissue divergences within a breed, indicating that gene expression regulation at transcriptional and translational levels is more conserved within the same tissue type across breeds than between different tissues within the same breed.

To investigate how translation modulates transcriptional differences between breeds, we quantified cross‐breed fold‐change variation at both the mRNA and RPF levels in five tissues (LW vs. BM pigs). We then examined whether translation modifies these transcriptional effects in a systematic manner by plotting mRNA log_2_FC against RPF log_2_FC (Figure 2d). Across all tissues, mRNA and RPF fold changes were positively correlated (*p* < 0.001), suggesting a general tendency for translation to reinforce transcriptional signals. Genes whose mRNA and RPF log_2_FC changed in the same direction were classified as reinforcing, whereas those with changes in opposite directions were classified as compensatory (Table S3). To evaluate the enrichment of these interaction types, we performed 1000‐iteration permutation analyses for each tissue (see Methods), revealing significant enrichment of compensatory and/or reinforcing interactions (*p* < 0.01) (Figure 2e; Figure S5). These results demonstrate that translation can either amplify or buffer transcriptional divergence between breeds. To further investigate how this post‐transcriptional regulation affects protein‐level differences between breeds, we integrated proteomic data (Table S4) and found that genes with reinforcing interactions exhibited greater inter‐breed protein divergence compared with compensatory genes (*p* < 0.05) (Figure 2f).

In summary, these findings highlight the pivotal role of translational regulation in reshaping transcriptional effects, thereby influencing final protein abundance and contributing to tissue‐ and breed‐specific phenotypic variation.

### 2.3 Dynamic Changes in TE as a Key Mechanism of Translational Regulation

**FIGURE 3:**
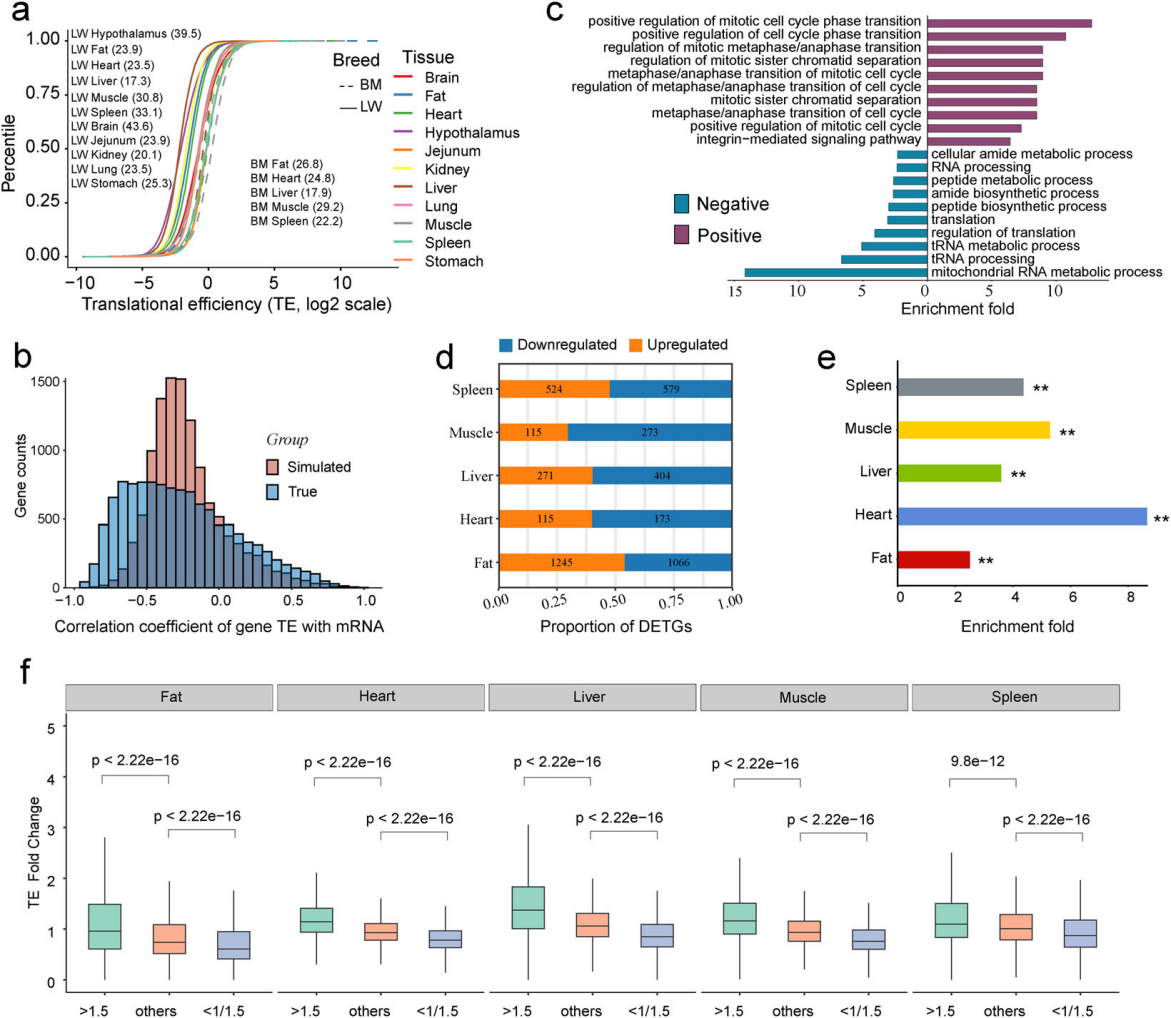
Translational efficiency dynamics across tissues and breeds. (a) Cumulative distribution of TEs of protein‐coding genes shared by all tissues across breeds (N = 11136). The TE range, defined as the ratio of the 97.5% to the 2.5% quantile of the TEs, was given for each tissue. (b) Gene TE and its correlation with gene mRNA expression across tissues. The blue bars represent the true correlation density distribution, while the red bars represent the simulated background, where the Ribo‐Seq read counts for different genes were randomly shuffled, and TE was recalculated using the randomized Ribo‐Seq counts in combination with the real RNA‐Seq read counts, and the RNA‐TE correlations are recalculated. (c) GO term enrichment analysis of the top 500 genes with positive and negative TE‐mRNA correlations. (d) Number and proportion of genes with upregulated and downregulated TE in each of the five tissues when comparing LW pigs to BM pigs. € Enrichment analysis of tissue‐specific DETGs within tissue‐wide DEGs was performed using the hypergeometric test, with ** meaning *p* < 0.01. (f) Relationship between the ratio of protein fold change to mRNA fold change and TE fold change in specific tissues across breeds. The *y*‐axis represents TE fold change between breeds, and the *x*‐axis shows groups based on the protein‐to‐mRNA fold change ratio: >1.5, <1/1/1.5, and others.

TE dynamics represent a central post‐transcriptional regulatory layer that modulates mRNA decoding and protein output, thereby shaping complex trait variation [58, 59]. To systematically characterize TE dynamics across tissues and breeds, we analyzed 11 136 protein‐coding genes with detectable TE in all examined tissues from both LW and BM pigs. TE distributions exhibited pronounced tissue specificity (Figure 3a; Kolmogorov–Smirnov test; *p* < 0.05). For instance, in LW pigs, the TE range spanned from 17.3‐fold in the liver to 43.6‐fold in the brain, indicating that broader TE ranges may confer increased flexibility for tissue‐specific translational regulation. TE‐range variability was generally more similar between breeds within the same tissue than across different tissues within the same breed, with the spleen being a notable exception (33.1‐fold in LW vs. 22.2‐fold in BM), potentially reflecting breed‐dependent differences in immune‐related translational programs. Moreover, TE distributions were consistently narrower than those of mRNA abundances across all tissues (Figure S6). This reduced variability likely reflects their distinct regulatory levels: mRNA abundance is subject to broad transcriptional fluctuations, whereas TE is constrained by the biochemical limits of ribosome recruitment and elongation, resulting in comparatively smaller dynamic ranges. Notably, we observed a significant negative correlation between gene‐level mRNA abundance and TE across tissues (Figure 3b; Two‐sample Kolmogorov–Smirnov test; *p* < 2.2e−16). A similar pattern has been reported in E. coli under diverse conditions, suggesting that this anticorrelation is a conserved regulatory strategy across both prokaryotes and eukaryotes, potentially serving to maintain protein synthesis homeostasis. GO enrichment analysis of the top 500 genes with the strongest positive and negative mRNA–TE correlations further revealed that positively correlated genes were enriched in cell cycle–related functions, whereas negatively correlated genes were associated with RNA metabolism, translational regulation, and protein synthesis (Figure 3c), highlighting the essential role of TE in sustaining gene expression and proteome homeostasis.

To evaluate how TE divergence between breeds contributes to phenotypic variation, we identified differential TE genes (DETGs) across five tissues in LW and BM pigs (|log_2_FC| > 1, FDR < 0.05) (Figure 3d; Table S5). We found that tissue‐specific DETGs were significantly enriched among tissue‐specific DEGs (*p* < 0.01; Figure 3e), underscoring TE's role in refining transcriptional outputs. GO analysis further revealed that DETGs were associated with tissue‐relevant pathways, including lipid metabolism in adipose tissue and energy metabolism in muscle (Figure S7), highlighting TE's contribution to breed‐specific physiological traits. To connect breed‐specific TE divergence with protein outcomes, we calculated the ratio of protein fold change to mRNA fold change between breeds. Genes with higher ratios tended to exhibit upregulated TE, while those with lower ratios showed downregulated TE (Figure 3f; *p* < 2.22e−16), emphasizing that TE substantially modulates protein output beyond transcript abundance.

Collectively, these findings establish TE as a dynamic and plastic post‐transcriptional regulatory mechanism. Variation in TE across tissues and between breeds contributes to physiological heterogeneity and phenotypic divergence by context‐dependently modulating protein abundance.

### 2.4 Diverse Factors Modulate Gene Translation Efficiency

**FIGURE 4:**
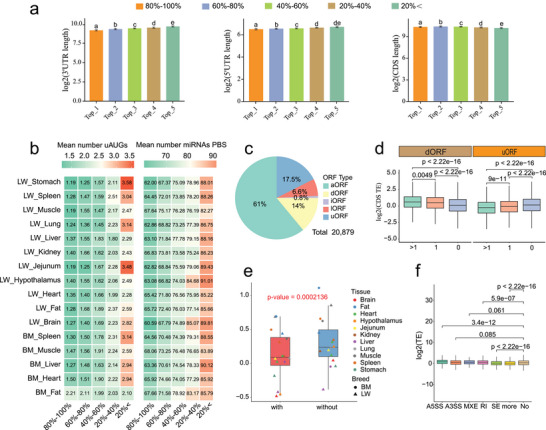
Determinants of translational efficiency. (a) Sequence lengths of 3’UTR, 5’UTR, and CDS for genes with different levels of CDS TE. Top 1–5 categories correspond to the 80%–100%, 60%–80%, 40%–60%, 20%–40%, and <20% percentiles based on the ranking of TE within each tissue. The median values for each percentile group across tissues are used for visualization. (b) The heatmap shows the average number of 5’UTR uAUGs (left) and 3’UTR miRNA PBSs (right) for genes with different degrees of TE. (c) Pie chart showing the proportion of different active ORF types detectable across all tissue samples in pigs. (d) Relationship between the number of active dORFs and uORFs and the TE of gene CDS. Statistical significance for inter‐group TE comparisons was determined using the Mann–Whitney U test. € Comparison of TE between genes with and without alternative splicing across tissues. *p*‐values were obtained using the Mann–Whitney U test. (f) Comparison of TE between genes of different alternative splicing types. “More” refers to genes with multiple AS events, “No” indicates genes without AS, and other specific AS names represent genes with only that particular AS type. *p*‐values were obtained using the Mann–Whitney U test.

As a deeper understanding of the mechanisms governing TE variation could provide valuable insights into the genetic basis of complex traits, we first evaluated the impact of sequence length of the 5’UTR, coding sequence (CDS), and 3’UTR on TE. Spearman correlation analysis revealed significant negative correlations between TE and both 5’UTR and 3’UTR lengths, while CDS length was positively correlated with TE (*p* < 0.05; Figure S8). Consistently, when genes were grouped into five percentile‐based TE categories (Top 1–5: 80%–100%, 60%–80%, 40%–60%, 20%–40%, <20%), genes with higher TE tended to have shorter 5’UTRs and 3’UTRs and longer CDSs (Figure 4a).

UTRs are well‐established cis‐regulatory elements that play crucial roles in maintaining mRNA stability and affecting translation [60, 61]. The upstream AUGs (uAUGs) within the 5’UTR may interfere with canonical start codons, thereby inhibiting translation [36, 62]. To examine this, we quantified uAUGs in 5’UTRs across TE categories and found that genes with higher TE contained fewer uAUGs. (Figure 4b; left). Motivated by this observation, we systematically identified actively translated open reading frames (ORFs) across all tissues (Figure S9a and Table S6). In total, 20 879 active ORFs were detected (Figure 4c), including 2923 dORFs in 3’UTRs, 3660 uORFs in 5’UTRs, 173 internal ORFs (iORFs) in annotated CDSs but in alternative reading frames, and 1386 ORFs in lncRNAs (lORFs). Analysis revealed that genes with more translated uORFs exhibited lower CDS TE, whereas genes with more dORFs showed higher CDS TE (Figure 4d), suggesting distinct mechanisms by which uORFs and dORFs fine‐tune translation. Consistently, protein‐coding genes rarely possessed both active uORFs and dORFs simultaneously (Figure S9b). Additionally, as miRNA binding to the 3’UTR is known to impact RNA stability and translation [63], we predicted miRNA binding sites in the 3’UTRs across TE categories and found that genes with higher TE had fewer 3’UTR miRNA sites (Figure 4b; right). Collectively, these results indicate that shorter UTRs enhance TE by minimizing inhibitory elements such as uAUGs and miRNA binding sites.

Alternative splicing (AS) is a major contributor to proteomic diversity [64], yet its impact on TE is less understood. In our study, we identified five major types of AS events—exon skipping (SE), mutual exon inclusion (MXE), alternative 3'splice site selection (A3SS), alternative 5'splice site selection (A5SS), and retained intron (RI)—across tissues from both breeds, totaling 94 579 AS events in 13 133 genes, with SE being most prevalent (Figure S10 and Table S7). We next compared the TE between genes with and without detectable AS events in each tissue. Genes undergoing AS exhibited significantly lower TE than those without AS (*p* < 0.05; Figure 4e). Examining specific AS types, genes with only SE events exhibited lower TE than those without AS, suggesting a repressive effect. In contrast, genes with A5SS or RI events showed higher TE, indicating promotive effects, while A3SS and MXE had no significant impact (Figure 4f).

In conclusion, we reveal the potential impact of gene sequence features and AS events on TE regulation. Importantly, these findings underscore the critical role of UTRs as cis‐regulatory elements in modulating translation, highlighting that genetic variation within these regions—particularly mutations affecting core regulatory elements—may profoundly influence translational outcomes.

### 2.5 Boosting Gene Regulatory Network Precision With Transcriptomic and Translatomic Integration

**FIGURE 5:**
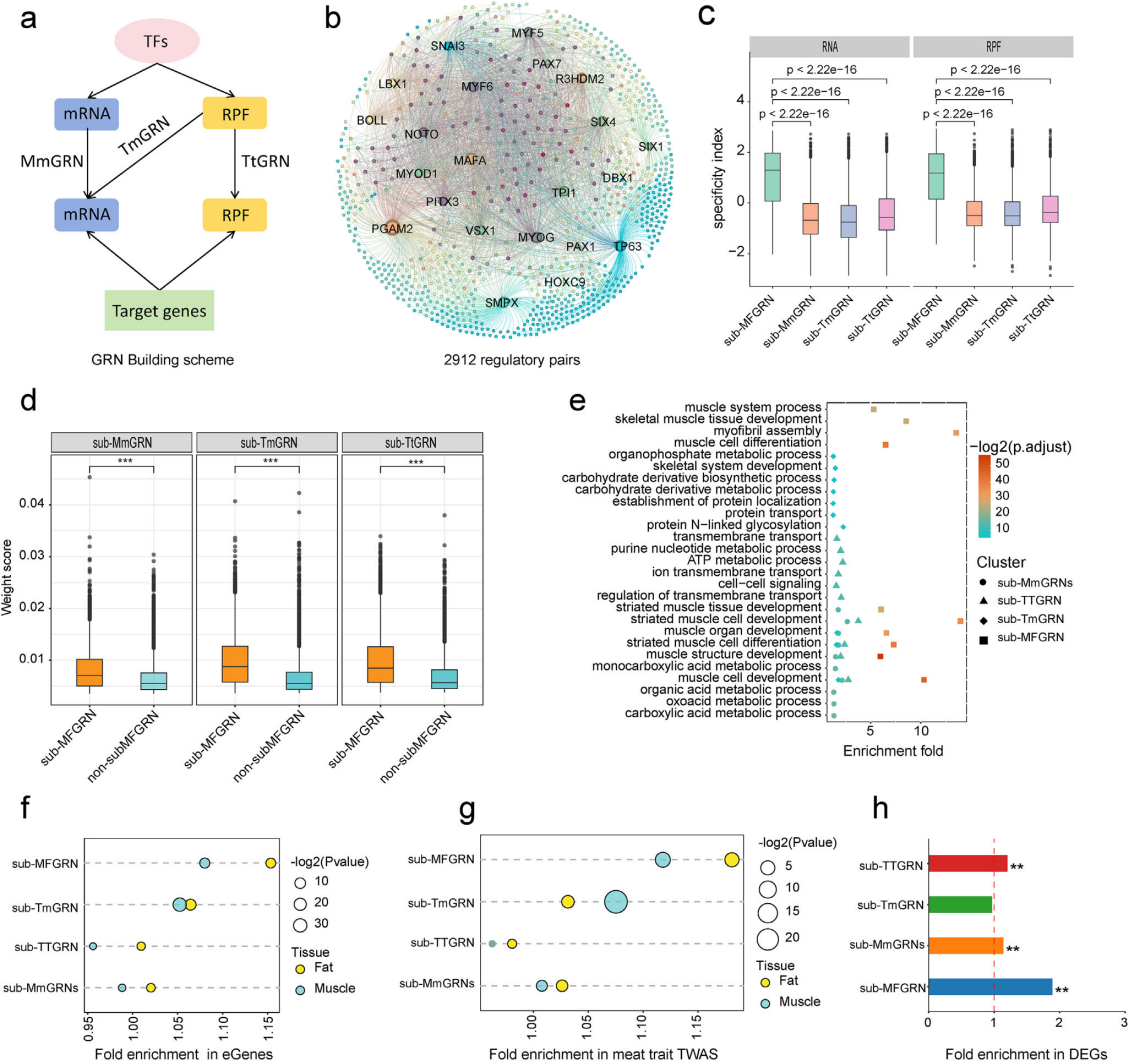
Translatome‐empowered dissection of trait‐specific GRN. (a) Schematic representation of different regulatory network construction models. (b) Visualization of the muscle tissue‐related MF GRN. (c) Comparison of muscle tissue‐specific expression indices for target genes of different types of GRNs in muscle tissue, at the RNA and RPF levels. Statistical significance was assessed using a Wilcoxon test. (d) Comparison of weight scores between target genes in the MF GRN and target genes in non‐MF GRNs across different GRNs. Statistical significance was assessed using a *t*‐test, with ** indicating *p* < 0.01 (e) Bubble plot showing the GO enrichment analysis results of target genes in the muscle tissue‐related MF GRN. (f) Enrichment analysis of target genes in muscle and adipose tissue‐related GRNs among corresponding tissue eGenes. Statistical significance was assessed using a hypergeometric test. (g) Enrichment analysis of target genes in muscle and adipose tissue‐related GRNs among genes identified in tissue‐specific trait‐associated TWAS analysis. Statistical significance was assessed using a hypergeometric test. (h) Enrichment of different type GRNs related to muscle tissue in DEGs. Significance was determined using the hypergeometric test, with ** indicating *p* < 0.01.

The dynamic regulation of TE buffers fluctuations in mRNA abundance, thereby influencing RPF levels and ultimately shaping protein output. This highlights the importance of integrating transcriptomic and translatomic data to elucidate regulatory relationships with greater precision. For instance, multi‐omics GRNs have successfully revealed functional landscapes in maize research [39]. Inspired by these advances, we investigated whether combining transcriptomic and translatomic data could enhance the identification of core regulatory networks that underpin pig complex traits. Using GENIE3 [65], we constructed three types of GRNs based on transcription factor (TF) and target gene expression matrices derived from all samples: intra‐omics GRNs within the transcriptome (MmGRNs) and translatome (TtGRNs), and inter‐omics GRNs (TmGRNs) (Figure 5a; for details, see Methods). Subsequently, by overlapping the top 1 million edges from these three GRNs, we constructed a high‐confidence multi‐omics fusion network (MF GRNs) (Figure S11a), encompassing 81 123 reliable TF‐gene regulatory relationships.

To assess whether the MF GRNs more effectively capture the genetic architecture underlying tissue‐specific physiological processes and traits, we focused on skeletal muscle and adipose tissues—two core tissues underlying meat production. Tissue‐specific TF regulators were then used to extract corresponding tissue‐specific sub‐networks from each GRN type, enabling a comparative evaluation of these sub‐networks' regulatory and functional capacities. We identified 22 tissue‐specific core TFs in skeletal muscle (such as *MYOD1*, *MYF5*, and *MYOG*) and 16 in adipose tissue (such as *CEBPA* and *PPARG*) (Figure S11b). Based on these TFs, we extracted four sub‐networks per tissue—sub‐MmGRN, sub‐TtGRN, sub‐TmGRN, and sub‐MF GRN. These sub‐networks encompassed 10 460, 9071, 15 455, and 2912 TF–target relationships in muscle, and 10 252, 7391, 12 891, and 1770 in adipose tissue, respectively (Figure 5b; Figure S11c). To explore the biological relevance of these sub‐GRNs, we performed GO enrichment analysis on the target genes in each network and visualized the top 15 enriched biological pathways. The sub‐MF GRNs showed the strongest enrichment for tissue‐specific biological processes in both muscle and adipose tissues (Figure 5e; Figure S12a). We further assessed the tissue specificity of target genes by comparing their RNA and RPF specificity indices between those included and excluded from the sub‐MF GRNs. Target genes in sub‐MF GRNs exhibited significantly higher tissue‐specific expression at both RNA and RPF levels (Figure 5c; Figure S12b), supporting their functional relevance in tissue‐specific biology. Furthermore, the weight scores of TF‐target gene pairs in MF GRNs were significantly higher than those in non‐MF GRN pairs within all three sub‐GRNs in both tissues (Figure 5d; Figure S12c), suggesting that the MF GRNs effectively reduce regulatory noise and enhance biological signal.

Given the importance of genetic variation in modulating complex traits through effects on gene expression, RNA stability, and TE, we next assessed the genetic explanatory power of the tissue‐specific sub‐GRNs using pig population genetics data. We first retrieved muscle‐ and adipose‐specific eQTL gene sets (eGenes) from the FarmGTEx database. Enrichment analysis showed that inter‐omics GRNs—particularly sub‐MF GRNs and sub‐TmGRNs—were significantly enriched for these tissue‐specific eGenes (*p* < 0.05, hypergeometric test), with sub‐MF GRNs exhibiting the highest enrichment fold (Figure 5f). We further evaluated the biological relevance of GRNs by comparing their associations with GWAS signals of meat production traits from the Animal QTLdb [66] (Table S8). While not all enrichments reached statistical significance, the sub‐MF GRNs consistently displayed the highest fold enrichment among the four GRN types (Figure S12d). To complement these results, we incorporated transcriptome‐wide association study (TWAS) data from the FarmGTEx database for meat production traits in muscle and adipose tissues (Table S9). Target genes in sub‐MF GRNs showed significant and specific enrichment in TWAS‐identified gene sets related to meat traits (Figure 5g). Finally, we assessed the overlap between sub‐MF GRN targets and DEGs between BM and LW pig breeds. Sub‐MF GRNs were significantly enriched for tissue‐specific DEGs in both muscle and adipose tissue, with higher enrichment than observed for the other three GRN types (Figure 5h).

In summary, MF GRNs offer a substantial advantage over single‐omics GRNs in elucidating the genetic architecture of complex traits. By integrating transcriptomic and translatomic data, they allow for more accurate identification of key regulatory genes and pathways, providing unique opportunities to identify critical cis‐regulatory variants affecting complex traits by modulating TE.

### 2.6 Unraveling the Regulatory Mechanisms of Trait by Refining the GRN Through Functional Experiments

**FIGURE 6:**
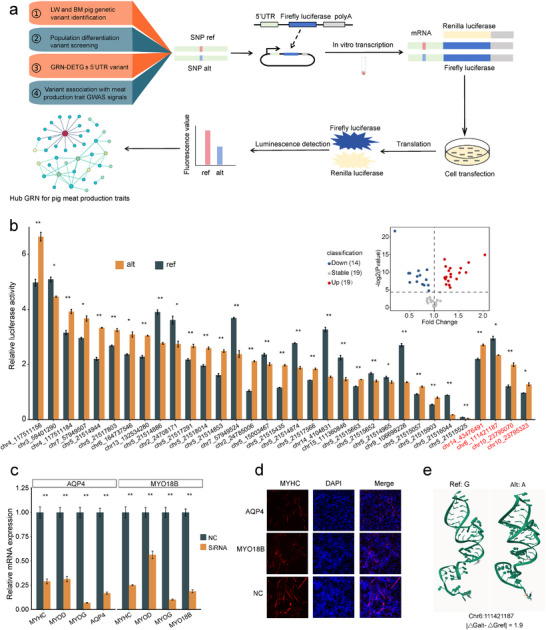
Translational regulatory variants refine GRN underlying traits. (a) Flowchart depicting the identification of functional variants in translation regulation related to pig meat production traits and the screening of the hub GRN. (b) Volcano plot illustrating the impact of 53 candidate SNPs in the 5’UTR region on gene TE. Bar plots display significant differences in luciferase activity for 33 SNPs with different alleles. ** denotes *p* < 0.01 and * denotes *p* < 0.05 by *t*‐test. Error bars represent mean ± SE (n = 3). Red labels on the x—axis indicate SNPs from muscle tissue, and black labels represent those from fat tissue. (c) qPCR analysis of the relative expression of myogenic markers in C2C12 cells with target gene knockdown during myogenic differentiation. Data are presented as mean ± SE (n = 3). Statistical significance was assessed using a *t*‐test, with ** indicating *p* < 0.01. NC, negative control. (d) Immunofluorescence staining of C2C12 myoblasts differentiated for 6 days. Myotubes were labeled with MyHC (red), and nuclei were counterstained with DAPI (blue). (e) mRNA conformation and free energy changes associated with different alleles of SNP Chr:111421187. |△Galt −△Gref| represents the difference in free energy between the mRNA sequences of the alternative and reference alleles.

Identifying regulatory variants associated with complex traits or diseases is essential for elucidating their genetic underpinnings. Consequently, fine‐mapping trait‐associated genetic loci plays a pivotal role in unraveling the mechanisms of trait inheritance. To further enhance the precision of the MF‐GRN, we incorporated population genetics and functional experiments to refine the GRN through the identification of translational regulatory variants (Figure 6a). We began by generating and analyzing WGS data from 24 BM and 22 LW pigs, which included all individuals used in this study. This analysis yielded 8 880 988 SNPs and 1 198 227 INDELs, among which we identified a subset with substantial genetic differentiation (ΔAF > 0.3) between the two populations (Figure S13a). We then focused on differentiated variants located in the 5’UTRs of DETGs from the sub‐MF GRNs—16 in muscle and 55 in adipose tissue—given the crucial role of the 5’UTR in regulating TE [61, 67, 68]. To further prioritize variants relevant to meat production, we retrieved 4829 SNPs associated with “Meat and Carcass Association” traits from the Animal QTLdb (Table S8). Candidate variants for functional validation were selected based on two criteria: (1) variants located within ±20 kb of relevant GWAS loci, or (2) variants not linked to GWAS loci but located within known regulators of tissue development.

These selected 5’UTR variants (9 in muscle sub‐MF GRN and 43 in fat sub‐MF GRN) were subjected to dual‐luciferase reporter assays to assess their effects on TE. Of these, 33 variants were confirmed to affect TE: 29 variants affecting 11 genes in adipose tissue and 4 variants affecting 3 genes in muscle tissue (*p* < 0.05; Figure 6b; Table S10). These translational regulatory 5’UTR variants of target genes further refined the hub GRNs that underlie the genetic basis of meat production traits (Figure 6a). To further investigate whether allelic differentiation aligned with functional effects, we examined whether the direction of TE changes observed in multi‐omics data and dual‐luciferase reporter assays corresponded with the allelic distribution between breeds. In adipose tissue, 15 variants across 6 genes (*VCAM1*, *ESYT1*, *CYP4B1*, *WNT10B*, *PIK3CG*, and *CSPG4*) showed consistent directional concordance; in muscle tissue, two variants across two genes also demonstrated this pattern (*MYO18B* and *AQP4*) (Figure S13b). To experimentally validate the robustness of this integrative approach, we selected *MYO18B* and *AQP4* for functional assays in C2C12 cells. Using RNA interference, we knocked down each gene and measured the expression of myogenic markers (*MYHC, MYOD*, and *MYOG*) by RT‐qPCR (Figure 6c). Immunofluorescence staining was also performed to assess the impact on myogenic differentiation (Figure 6d). Both assays consistently showed that *MYO18B* and *AQP4* promote myoblast differentiation, supporting their functional roles in skeletal muscle development. To explore the underlying mechanisms by which the identified functional variants regulate translation, we utilized the FIMO tool [69] and RNAfold software [70] to assess how different alleles of functional variants affect RNA‐binding protein (RBP) interactions and RNA secondary structure. The results suggest that variants in *MYO18B* (chr14_43476491) likely modulate TE through altered RBP binding (Table S11), while the variant in *AQP4* (chr6_111421187) may influence RNA structural conformation (Figure 6e).

In summary, by integrating multi‐omics GRNs with population genetics and experimental validation, we established a systematic framework for constructing hub GRNs that elucidate the genetic and regulatory mechanisms underlying meat production traits in pigs.

## 3 Discussion

In this study, we developed an optimized strategy for constructing gene regulatory networks by integrating transcriptional and translational profiling, genetic differentiation, GWAS, and systematic functional validation. This integrative approach enables a more comprehensive understanding of the genetic mechanisms underlying complex traits from a translational perspective. Using pigs as a model, we demonstrated that incorporating gene‐level translational signals into GRN construction significantly enhances the network's ability to explain the genetic basis of complex traits. Furthermore, by refining MF GRNs to retain only functional 5’UTR variants supported by both population genetics and experimental evidence, we identified hub GRNs that are more likely to influence complex traits. Within these networks, we pinpointed translation‐modulating variants in the 5’UTRs of target genes associated with trait regulation. This framework addresses a long‐standing gap in the field by introducing translational regulation as a critical and previously underappreciated layer in the dissection of genotype–phenotype relationships.

In our study, we observed that only genes with high transcriptional activity produced detectable protein signals in proteomic analyses (Figure S14b). Across different tissues, the proportion of genes with detectable protein levels ranged from 28% to 53% (Figure S14a), highlighting the current sensitivity limitations of proteomic technologies and the challenges they pose for large‐scale applications. Notably, our results indicate that RPFs may serve as more reliable proxies for protein abundance than mRNA levels (Figure S3; Figure 1h). Given the critical role of translational regulation in shaping protein output, it represents a key regulatory layer for understanding the genetic architecture of complex traits. As such, the multi‐tissue translational profiling generated in pigs offers valuable resources for more precise and comprehensive dissection of the molecular mechanisms underlying complex traits.

Dynamic changes in gene expression contribute to the foundation of adaptive evolution of complex phenotypes [71, 72]. In our study, we observed significant differences in gene expression variability between mRNA and RPF levels, both within and between tissues (Figures 2b,c). This phenomenon has been documented in previous studies of both rice [58] and mouse [37], highlighting the pivotal role of post‐transcriptional translational regulation in shaping phenotypic diversity across species. Interestingly, we found that although mRNA and RPF tissue‐specific genes are enriched in distinct functional pathways (Figure S15a), both are strongly linked to the biological processes specific to their respective tissues (Figure S15c,d) Moreover, tissue‐specific expression indices at both the mRNA and RPF levels are strongly conserved between pigs and mice (Figure S15b), further highlighting the cross‐species conservation of transcriptional and translational dynamics and underscoring the importance of integrating transcriptomic and translational data to dissect complex traits. Through breed‐specific differential expression analysis of RNA and RPF levels across tissues, we identified significant enrichment of compensatory and/or reinforcing interaction genes in each tissue (Figure 2e; Figure S5), which contributed to breed‐specific protein abundance differences (Figure 2f). This revealed translation buffering and amplification as predominant forms of post‐transcriptional regulation [37, 73, 74, 75]. These findings illustrate the limitations of relying solely on eQTL analyses to account for variation in protein abundance and further emphasize the necessity of integrating both transcriptomic and translational data [20, 21, 31, 32]. Notably, cross‐tissue PCC analysis (Figure S16a–e) showed that, across compensatory and reinforcing genes, as well as other gene sets, RNA and RPF variation were positively correlated in most genes across tissues, indicating that translational regulation primarily modulates the magnitude of transcriptional effects, rather than reversing their direction.

To prioritize critical regulators underlying complex traits, we integrated both transcriptomic data and translational data to construct GRNs. While numerous multi‐omics‐based GRN frameworks have been developed and successfully applied to elucidate the genetic basis of complex traits, most have focused primarily on integrating transcriptomic and epigenomic data to characterize gene regulation at the transcriptional level [6, 76, 77, 78]. In contrast, our study underscores the critical role of translational control in post‐transcriptional regulation and demonstrates that incorporating translatomic data can effectively reduce noise and improve the resolution of regulatory relationships. Consistent with this, previous work by Zhu et al. in maize demonstrated that integrating transcriptomic and translatomic data into GRNs enables a more precise identification of the core regulatory networks that govern phenotypic traits [39]. Building on this, we used our integrated GRN as a foundation and combined it with GWAS signals, genetic differentiation analysis, and luciferase reporter assays to systematically identify phenotype‐modulating variants that act through translational regulation. This integrative framework successfully uncovered key translation‐modulating variants and their target genes, substantially broadening the scope beyond variants that influence RNA abundance alone. Therefore, our approach offers a powerful and generalizable strategy for uncovering genetic regulators of complex traits that may have been previously overlooked.

While edge‐overlap‐based GRN strategies provide a robust and interpretable framework for network inference, our analysis also identified a subset of compensatory genes whose mRNA variation is buffered at the translational level, manifested as discordant changes between mRNA and RPFs (Figure 2d). These cases likely reflect scenarios in which a weaker selective constraint on transcript abundance allows subtle transcriptional regulatory shifts that are subsequently compensated during translation, often resulting in apparently reversed regulatory signals. Genes governed by such decoupled transcriptional and translational control may therefore be incompletely represented in our inferred network. Importantly, this observation does not diminish the utility of edge‐overlap‐based approaches, but rather highlights a broader challenge and important future direction in modeling multilayer regulatory architectures, particularly for genes whose protein abundance is stabilized through translational buffering.

In our luciferase reporter assays, 16 of the 33 tested variants exhibited allelic effects that were discordant with allele‐specific TE signals. Although luciferase reporter assays are widely used to identify variants that modulate the activity of cis‐regulatory elements, discrepancies between reporter assay–based measurements and endogenous molecular phenotypes are well documented. For instance, concordance rates between enhancer or promoter activities measured by reporter assays and eQTL effects typically range from ∼48% to 80%, depending on the epigenomic context of the variants [79]. To our knowledge, no systematic comparison has been performed between allelic effects measured using 5’UTR luciferase assays and allelic differences detected by TE. Our observed concordance rate of 51.5% falls within this previously reported range, and its position near the lower end likely reflects the stronger context dependence of translation efficiency, which is more easily disrupted when reporter constructs lack the endogenous coding region and 3’UTR. While acknowledging the inherent limitations of luciferase reporter assays and the presence of discordant cases, we used luciferase assays primarily as a prioritization filter—focusing downstream functional validation on variants supported by multiple orthogonal lines of evidence, including concordant effect directions between luciferase assays and allelic differences in TE. This concordance‐based prioritization strategy has also been adopted in several prior studies [6, 78, 80, 81].

In our study, we observed a general trend of negative correlation between mRNA abundance and TE, suggesting that TE serves as a key regulatory mechanism in fine‐tuning gene expression output. Interestingly, this relationship exhibits distinct biological signatures. Genes with a high negative correlation between mRNA and TE were significantly enriched in pathways related to gene translation and protein synthesis, while genes showing a positive correlation were notably enriched in cell cycle‐related pathways. Since essential genes are typically central to core cellular processes and highly conserved [82], this pattern implies that TE plays a critical role in preserving the functional stability of essential genes. The positive correlation with cell cycle pathways may reflect the need for precise regulation to ensure efficient cell division and growth. In contrast, the negative correlation likely represents a protective mechanism to balance resource consumption and preserve cellular function, preventing excessive energy expenditure in essential biological processes. Consequently, the control of TE plays a pivotal role in shaping specific biological processes and the formation of complex phenotypes. Our analysis further reveals that the regulation of gene TE is complex and influenced by multiple factors, including gene sequence length [42], molecular features of the 5’UTR [83, 84] and 3’UTR [85] regions, and AS events [86]. Specifically, we found that CDS TE is negatively correlated with the sequence lengths of both the 5’UTR and 3’UTR. This is likely due to the increased presence of cis‐regulatory elements in longer UTRs, such as the number of uAUGs in the 5’UTR and RBP sites in the 3’UTR, which can modulate CDS TE through distinct mechanisms. Consistent with observations by Xie et al. in mouse tissue [37], where AS was associated with reduced TE, our results in pigs show a similar trend (Figure 4e). Notably, while the most common splicing event, SE, is generally associated with lower TE, other AS types exhibit distinct effects. For example, genes with A5SS or RI events display higher TE, suggesting a potential promotive role in translation (Figure 4f). Similarly, Qin et al. reported that trans‐splicing enhances TE in C. elegans [86]. These findings indicate that the impact of AS on TE likely depends on the specific type of splicing event, and the precise causal mechanisms underlying these effects warrant further investigation. Together, these results offer a comprehensive view of the mechanisms shaping TE and underscore its significance in post‐transcriptional gene regulation.

In addition to systematically investigating translational regulation at the gene level, this study also refines the translational annotation of the pig genome across tissues and breeds at the ORF dimension. This includes canonical translation events (aORFs and iORFs) as well as cryptic non‐canonical ORFs (uORFs, dORFs, and lORFs). We first analyzed the sequence lengths (Figure S9c) and activity characteristics (Figure S9d) of different ORF types. Given that cryptic non‐canonical ORFs have emerged as an important topic in translational regulation research in recent years [87, 88, 89], we conducted an in‐depth analysis of these elements. We found that non‐canonical ORFs generally exhibited lower transcription–translation correlations (r) compared to canonical ORFs (Figure S9e), suggesting that non‐canonical ORFs may be subject to more complex post‐transcriptional regulatory mechanisms. Meanwhile, the TE of these non‐canonical ORFs showed higher tissue specificity (Figure S9d), and these tissue‐specific ORFs may play important roles in the regulation of tissue‐related biological processes (Figure S17b). uORFs are well known for modulating mRNA translation in eukaryotes [90], typically inhibiting downstream gene translation by sequestering ribosomes from the CDS [68, 83, 84, 91, 92, 93, 94, 95, 96, 97]. Previous studies in humans, mice, and zebrafish have shown that the number of uORFs correlates with the extent of translation inhibition of the canonical ORF [98, 99], and our findings in pigs further support this cross‐species pattern (Figure 4d). Additionally, Wu et al. found that dORFs enhance canonical ORF TE, and the number of dORFs in human genes is positively correlated with canonical ORF TE [100]. Similarly, Chen et al. demonstrated that dORFs enhance gene TE in heat‐treated grains [101]. Our study in pigs also reveals a positive correlation between the number of dORFs and the TE of upstream CDS (Figure 4d), suggesting that dORFs’ regulatory role in modulating TE may be a conserved mechanism across species. Interestingly, we observed that dORFs and uORFs rarely co‐occur in the same gene (Figure S9b). Furthermore, genes harboring dORFs and uORFs are enriched in distinct biological processes (Figure S17c), indicating a functional distinction between these two types of ORFs. This mutually exclusive pattern suggests that genes tend to adopt one of two regulatory strategies: one driven by dORFs, which promotes faster, more responsive translation, and another driven by uORFs, which maintains a lower, more tightly regulated level of translation. This functional specialization likely enhances gene expression efficiency, enabling better adaptation to changing cellular demands or environmental conditions. In addition, existing evidence has shown that lORFs can encode small peptides [38, 102, 103, 104], which may play a pivotal role in regulating biological processes [103, 105, 106]. In our study, we identified 1386 lORFs across different pig tissues, demonstrating strong tissue specificity (Figure S17a). Notably, we also observed that lncRNAs producing active ORFs exhibit higher expression levels compared to those without active ORFs (Figure S17d), a finding previously reported in Drosophila [107]. The actively translated lORFs identified in this study serve as a candidate set for further validation of small peptide production and their functional roles in regulating key biological processes in pigs. In conclusion, this work provides valuable data resources for future studies investigating the genetic basis of complex traits in pigs from the ORF perspective.

In summary, our study has compiled a comprehensive dataset encompassing gene expression and translation in terms of protein abundance across multiple tissues from both Western and Eastern pig breeds. These data provide valuable insights into the post‐transcriptional translation regulation that governs phenotypic differences in pigs. Furthermore, we optimized the GRN construction strategy, thereby establishing a paradigm for elucidating the genetic regulatory mechanisms underlying complex traits from a translational perspective.

## 4 Methods

### 4.1 Pig Tissue Collection

In this study, tissue samples were collected from 21‐day‐old piglets of two breeds: the Chinese local breed BM and the Western commercial breed LW. Three individuals from each breed were sampled as biological replicates. A total of 11 tissues were collected from LW pigs, including the Heart, Fat, Liver, Kidney, Muscle, Hypothalamus, Jejunum, Stomach, Brain, Spleen, and Lung. For BM pigs, samples were collected from five tissues: Heart, Liver, Muscle, Fat, and Spleen. All fresh samples were promptly frozen in liquid nitrogen and stored at −80°C until further use. All experimental procedures were approved by the Ethics Committee of Agricultural Genomics Institute at Shenzhen (AGIS‐ER‐2024‐003).

### 4.2 Library Preparation and Sequencing

For Ribo‐seq, every tissue samples were lysed in lysis buffer (containing polysome buffer, Triton X‐100, DTT, DNase I, cycloheximide, NP‐40, and nuclease‐free water). The lysate was incubated on ice for 20 min and then clarified by centrifugation at 20 000 × g for 3 min at 4°C. For each sample, 10 µL RNase I (NEB, M0307) and 6 µL DNase I (NEB, M0303) were added and incubated for 45 min at room temperature. Nuclease digestion was halted by adding 10 µL of SUPERase·In RNase inhibitor (Invitrogen, AM2694). Equilibration of size exclusion columns (Merck KgaA, 27‐5140‐01) was performed with 3 mL polysome buffer under gravity flow, centrifuged at 600 × g for 4 min at room temperature. Then digested products from the last step were added to the prepared Size exclusion columns and centrifuged at 600 × g for 4 min at room temperature. Next, 10 µL 10% (wt/vol) SDS was added to the elution, and RFs with a size greater than 17 nt were isolated according to the RNA Clean and Concentrator‐25 kit (Zymo Research, R1017). rRNA was removed using the method reported previously [108]. Finally, RFs were further purified using magnet beads (Vazyme, N412‐01). For library construction, adapters were added to both ends of the RFs with NEBNext Multiple Small RNA Library Prep Set for Illumina (NEB, E7300L), followed by reverse transcription and PCR amplification to enrich for PCR products in the 140–160 bp size range. The resulting DNA library was sequenced on an Illumina NovaSeq X Plus platform by Gene Denovo Biotechnology Co. (Guangzhou, China).

For RNA‐seq, Total RNA was extracted and used as input for RNA‐seq sample preparation. rRNA was removed using specific probes. The RNA was then fragmented using divalent cations under elevated temperature in the First Strand Synthesis Reaction Buffer. First‐strand cDNA was synthesized using random hexamer primers and reverse transcriptase. The second strand was synthesized by adding buffer and dNTPs, with dTTP replaced by dUTP. The synthesized double‐stranded cDNA was subjected to terminal repair, A‐tailing, adapter ligation, and PCR enrichment following fragment size selection. For size selection, cDNA fragments of 370–420 bp were purified using AMPure XP beads to obtain strand‐specific libraries. The quality of the library was assessed by preliminary quantification using Qubit. Once the fragment size distribution was verified, the effective concentration of the library was accurately quantified by qRT‐PCR to ensure quality. Qualified libraries were pooled and sequenced on Illumina platforms, according to the required concentration and data output specifications.

### 4.3 Sequencing Data Preprocessing

For RNA‐seq, raw sequencing data were quality‐controlled using Trim Galore (version 0.6.10) with the parameters ‐q 20 –length 25 −e 0.1. High‐quality reads were aligned to the pig reference genome (Sscrofa11) using HISAT2 (version 2.2.1) [109] with the –rna‐strandness RF option. Gene‐level counts were obtained from properly aligned paired‐end reads using featureCounts (version 2.0.2) [110] with the parameters ‐B ‐C ‐p ‐s 2 –countReadPairs. To identify alternative splicing events, the rMATS (version 4.3.0) [111] software was employed, processing BAM files derived from the genome alignments of each tissue. High‐confidence alternative splicing events were then filtered using the criteria of IJC_SAMPLE_1> 10 or SJC_SAMPLE_1> 10 for each tissue.

For Ribo‐seq, raw Ribo‐seq data underwent quality control with fastp (version 0.23.4) [112] using the parameters ‐q 20 ‐c –length_required = 20 –n_base_limit 5. Reads mapping to rRNA and tRNA were filtered out using Bowtie2 (version 2.5.1) [113] with the –un‐gz option. The remaining high‐quality reads were aligned to the pig reference genome (Sscrofa11) using STAR (version = 2.7.10b) [114], and gene‐level counting was performed with featureCounts using the parameter ‐s 1. To account for differences in library composition across samples, raw counts from all RNA‐seq and Ribo‐seq samples were combined and normalized using a pool‐based size factor calculated with the DESeq2 (version1.34.0) [115] R package. The normalized counts were then converted into transcripts per kilobase million (TPM) values for further analysis. In our study, genes were considered detectable at a given level (either RNA‐seq or Ribo‐seq) in a tissue if the total reads across the three biological replicates exceeded 10 and the gene was detected in at least two of the samples.

### 4.4 Ribo‐seq Signal Assessment

The riboWaltz (version 1.2.0) [116] software was used to evaluate the distinct signal features of ribosome profiling data. RPF lengths were determined using the bamtolist() function, and fragments with lengths ranging from 20 to 35 nt were selected for downstream analysis using the length_filter() function. The P‐site offset for each read length was calculated with the psite() function. The proportion of footprints corresponding to the primary reading frames was calculated using the frame_psite() function, and codon periodicity was assessed with the metaprofile_psite() function. The results from these analyses were visualized using custom R scripts. To assess the biological reproducibility of different tissue samples, PCA was performed on the normalized RPF expression matrix for all samples using the prcomp function in R. The PCA results were then visualized using the Scatterplot3d (version 0.3.44) package in R.

### 4.5 DIA‐Based Proteomics Experimental and Analytical Method

Extract total proteins using the lysis buffer and precipitate total proteins using cold acetone. Digest the total proteins overnight with Trypsin (Promega, V5113). Peptides were separated on an XBridge C18 column (4.6 mm x 250 mm, 5 µm, Waters Corporation,186003117) using an Ultimate 3000 system (ThermoFisher Scientific, MA, USA) with a 40‐min gradient (5%–45% buffer B). Six fractions were collected and dried. Peptides were analyzed on an EASY‐nLC 1200 system coupled to an Orbitrap Lumos mass spectrometer (Thermo Fisher Scientific, MA, USA). Raw data were processed using Spectronaut X (Biognosys AG) with the Uniprot database. Fixed carbamidomethylation (C) and variable oxidation (M) were specified. A 1% FDR was applied at precursor and peptide levels. Samples were resuspended in 0.1% formic acid, mixed with iRT peptides, and analyzed using the EASY‐nLC 1200 system (Thermo Fisher Scientific, MA, USA) and Orbitrap Lumos. Peptides were separated on an Acclaim PepMap C18 column with a 120‐min gradient (5%–35% buffer B). Variable window acquisition was performed with 60 overlapping windows (1m/z overlap). Proteins were identified by matching DIA data to the spectral library (1% FDR at precursor and protein levels). Quantification was based on the average peak area of the top three MS1 peptides (FDR < 1.0%), with local normalization applied using Pulsar software.

### 4.6 Identification of LncRNAs

To systematically identify genomic lORFs, we employed the following pipelines to identify novel lncRNAs (Figure S18). RNA‐seq BAM files were processed using StringTie (version 2.2.1) [117] for transcript assembly, employing both de novo and reference‐guided strategies to construct a comprehensive transcriptome. Single‐exon transcripts were filtered using gffcompare, and transcripts located in intergenic regions were identified with inhouseGTFtools by applying the ‐classCode u parameter. Transcripts shorter than 200 bp were excluded, followed by coding potential analysis using CNCI, which retained only non‐coding candidates. To further refine the dataset, hmmscan was utilized to screen all six reading frames for homologous sequences against the Pfam protein family database, and transcripts with significant matches (E‐value < 1 × 10^−5^) were removed. Additionally, BLASTx (version 2.11.0+) [118] was used to exclude transcripts with significant similarity to known proteins in the NCBI NR and UniRef90 databases (E‐value < 1 ×10^−5^). Finally, only transcripts expressed in at least one sample were retained, ensuring a stringent and reliable identification of lncRNA candidates for subsequent analyses.

### 4.7 Evaluation of Gene Expression Diversity

To systematically compare gene expression variation between RNA and RPF within tissues, we conducted variance analysis on 12 849 genes with detectable transcriptional expression across all tissues. Variance for both RNA and RPF levels was calculated in each tissue using the var() function in R. Subsequently, we performed Wilcoxon rank‐sum tests to compare variation in RNA and RPF within tissues of specific breeds, as well as the variation in RNA and RPF between breeds. Additionally, to compare gene expression divergence across tissues and breeds, we applied the Euclidean distance metric to measure gene expression differences between pairs of tissues within specific breeds, as well as between breeds within specific tissues. The Euclidean distance (also referred to as the root mean squared deviation) is calculated using the following formula:

$$D\left(x,y\right)=\sqrt{\frac{1}{N}\sum_{i=1}^{N}{\left(\log_{2}\left(x_{i}+1\right)-\mathrm{lo}g_{2}\left(y_{i}+1\right)\right)}^{2}}$$

x_i_ and y_i_ represent the normalized TPM values of gene i in two samples from tissues n and m, respectively. *N* denotes the total number of protein‐coding genes considered in the global gene expression comparison (here, *N* is 11 136). Notably, for each tissue pair, there were nine possible sample combinations, accounting for three replicates per tissue. A higher Euclidean distance value indicates greater divergence between the tissues, reflecting a more pronounced dissimilarity in their gene expression profiles. For the inter‐group comparisons, the Wilcoxon rank‐sum tests were performed using the ggsignif package in R, and the results were visualized using the ggplot2 (version 3.5.1) [119] package in R.

### 4.8 Tissue‐Specificity Analysis of Gene Expression

To investigate the biological functions of tissue‐specific genes at different molecular levels, we further selected tissue‐specific genes based on their tissue specificity index (TSI), using the method described previously [78]. Genes with a TSI greater than 0.9 were considered to have tissue‐specific expression. The GO enrichment analysis conducted in this study was performed using the enrichGO function from the clusterProfiler (version 3.14.3) R package [120].

### 4.9 Differential Gene Expression Analysis

DEGs between LW and BM pigs within specific tissues were identified using the DESeq2 [115] (version 1.34.0) R package. Raw read counts for genes detected in each tissue were used as input for differential expression analysis. Genes were considered differentially expressed if they met the criteria of FDR < 0.05 and |log2FoldChange| > 1. DETGs between LW and BM pigs within specific tissues were identified using the Xtail (version 1.1.5) [121] R package. For this analysis, RNA‐seq and Ribo‐seq counts from the gene CDS regions, quantified using featureCounts, were used as input. DETGs were then selected based on the criteria of FDR < 0.05 and |log2FoldChange| > 1.

### 4.10 Significance Testing of Compensatory and Reinforcing Interactions

To evaluate how transcriptional and translational changes interact across breeds, we performed a permutation‐based enrichment analysis in each of the five tissues. For every gene, we calculated the log_2_FC in mRNA and RPF abundance between BM and LW pigs. Genes were then assigned to two interaction categories: reinforcing, when mRNA and RPF log_2_FC had the same sign and fell beyond two standard deviations along the line y = x; and compensatory, when the two log_2_FC values had opposite signs and exceeded two standard deviations along y = –x. A filter (|log_10_(|mRNA_log_2_FC/RPF_log_2_FC|)| < 0.5) was applied to ensure both measurements contributed meaningfully. To test whether these interaction types were enriched, we generated 1000 randomized datasets for each tissue. In each iteration, the observed mRNA log_2_FC values were paired with a permuted RPF log_2_FC set (excluding self‐pairing), which was then scaled to match the original mean, standard deviation, and correlation with mRNA log_2_FC. We applied the same classification to each randomized dataset to obtain null distributions for the number of reinforcing and compensatory genes. Empirical *p*‐values were calculated by comparing observed counts with the null expectations, with significance defined as *p* < 0.01.

### 4.11 Identification of Active ORFs

The pig reference genome annotation file (Sus_scrofa.Sscrofa11.1.110.gtf) and an integrated dataset of identified lncRNAs were employed to systematically identify unannotated translated ORFs across the pig genome. The analysis leveraged ribosome profiling data processed with RiboCode (version 1.2.13) [122], a specialized tool for detecting translated ORFs. Initially, transcript annotation files were prepared using the prepare_transcripts function with default settings. The offset parameters, representing inferred P‐site positions for varying ribosome footprint lengths, were subsequently determined using the metaplots function with the ‐i parameter. Offset values for ribosome footprints of 25 to 32 nucleotides were calculated as 12, 12, 11, 12, 12, 12, 12, and 12, respectively. To identify translated ORFs, merged BAM files from biological replicates were processed using RiboCode and the defined offset parameters. ORFs encoding peptides of at least eight amino acids were retained. The resulting ORFs were assigned to five categories: aORFs, which overlap with the annotated CDS and share the same stop codon; uORFs, located upstream of the annotated CDS without overlap; dORFs, located downstream of the annotated CDS without overlap; iORFs, situated within the annotated CDS but translated in an alternative reading frame; and lORFs, which overlap with annotated lncRNAs.

To identify actively translated ORFs in each tissue, featureCounts was used to quantify RPF and RNA reads aligned to ORFs based on the GTF file generated by RiboCode and BAM files from tissue‐specific translatome and transcriptome alignments. For transcriptome data, the parameters were configured as ‐B ‐C ‐p ‐s 2 –countReadPairs ‐g orf_id ‐t exon, while for translatome data, the parameters were set as ‐d 25 ‐D 32 ‐s 1 ‐g orf_id ‐t exon. An ORF was classified as actively translated in a given tissue if it met the following criteria across three biological replicates: (1) the total read count at both the transcriptional and translational levels exceeded 10, and (2) at least two biological replicates at both levels had detectable read counts. For normalization, the count matrices of transcriptional and translational levels for each tissue were processed using a pool‐based size factor calculated with the DESeq2 R package. The normalized counts were subsequently converted into TPM values to facilitate downstream analyses.

### 4.12 Evaluation of Translational Efficiency Dynamics

Genes exhibiting detectable RNA and RPF signals within a given tissue were defined as TE‐detectable genes. Using 11 136 protein‐coding genes with detectable TE signals across all tissues (with TE detectability defined as meeting detection thresholds for both mRNA and RPF), we assessed variability in the distributions of TE and RNA expression across tissues and breeds. TE distribution within each tissue was quantified by calculating the ratio of the 0.975th to the 0.025th percentiles of gene‐level TE values after sorting genes by TE. Similarly, RNA expression distribution was evaluated by calculating the ratio of the 0.975th to the 0.025th percentiles of RNA expression values after sorting genes by RNA abundance. Cumulative distribution curves of ranked TE values for each tissue were visualized using ggplot2. To quantify the relationship between TE and RNA abundance, we calculated the Pearson correlation between TE and RNA for TE‐detectable genes across 11 tissues in Large White pigs using the cor.test function in R. To further validate the correlation distribution, we performed a permutation analysis by randomly shuffling Ribo‐seq read counts across genes and recalculating TE using the permuted Ribo‐seq counts combined with the real RNA‐seq read counts. Differences between the observed and simulated correlation distributions were evaluated using the asymptotic two‐sample Kolmogorov–Smirnov test. Correlation distributions from both real and permuted datasets were visualized as bar plots generated with the ggplot2 (version 3.5.1) package.

### 4.13 Determinants of TE

To assess the impact of sequence length on gene TE, we selected the transcript with the highest expression level (TPM > 0.01) as the representative for each gene. We then calculated the correlation between the sequence lengths of the 3’UTR, 5’UTR, and CDS regions and gene TE in each tissue using the R function cor.test (method = “spearman”). Subsequently, we calculated the percentiles of TE values within each tissue. TE was categorized into five groups based on the 0%–20%, 20%–40%, 40%–60%, 60%–80%, and 80%–100% percentiles, designated as Top1, Top2, Top3, Top4, and Top5, respectively. For each tissue, we computed the average sequence length for each TE group and performed statistical significance analysis between groups using the Wilcoxon test (wilcox.test) via the ggsignif R package. The bedtools getfasta function was used to extract the 5’UTR and 3’UTR sequences of the transcripts. To compare the number of upstream AUG (uAUG) sites in the 5’UTRs of genes with different translation efficiencies, we utilized a Python script to count the uAUGs in the 5’UTR sequences of each transcript. To compare the number of miRNA binding sites (PBS) in the 3’UTRs of genes with varying translation efficiencies, we retrieved the 914 pig miRNA sequences from miRBase [123]. Using the miRanda software [124], we performed miRNA PBS scanning on the 3’UTR sequences, and an R script was employed to calculate the number of miRNA PBS sites in the 3’UTR of each transcript. The average number of miRNA PBS sites in the 3’UTR and the average number of uAUG sites in the 5’UTR for each TE group were visualized. All visualizations were generated using the ggplot2 package in R.

### 4.14 Whole Genome Sequencing (WGS) Data Analysis

In this study, we analyzed WGS data from 24 BM pigs and 22 LW pigs, including data from 3 BM and 3 LW pigs generated in‐house, with the remaining data obtained from public datasets. Quality control of the WGS data was performed using fastp, followed by alignment with BWA. Duplicate reads were marked using GATK (version 4.1.4.1) [125] MarkDuplicates with default parameters. Genome variant calling was performed for each sample using GATK HaplotypeCaller, and variant calls across all samples were subsequently combined. Genotyping was conducted using GATK GenotypeGVCFs. Variant types (SNPs/INDELs) were selected using GATK SelectVariants. For SNPs, variants were filtered using the VariantFiltration tool with the following criteria: QUAL < 30.0, QD < 2.0, FS > 60.0, MQ < 40.0, SOR > 3.0, ReadPosRankSum < −8.0, and MQRankSum < −12.5 to exclude low‐quality SNPs. For INDELs, variants were filtered with the following criteria: QUAL < 30.0, QD < 2.0, FS > 200.0, SOR > 10.0, ReadPosRankSum < −20.0, and MQRankSum < −12.5 to exclude low‐quality INDELs.

To perform population differentiation analysis, variants were further filtered using bcftools (version 1.14) [126] filter with the criteria “MAF >= 0.05 && F_MISSING <= 0.2”. bcftools view was used to extract variant information for the BM and LW populations separately. The bcftools fill‐tags command was then employed to regenerate the annotation information for the BM and LW VCF files. Finally, bcftools query was used to extract the reference allele frequency for each population. Variants with a reference allele frequency greater than 0.3 in both BM and LW populations were considered population‐differentiated variants.

### 4.15 Establishment of GRNs

The set of genes detectable at both the transcriptional and translational levels in any tissue (n = 17 206) was used to construct gene regulatory networks (GRNs). The TFs list was sourced from this resource (https://github.com/aertslab/pySCENIC/blob/master/resources/hs_hgnc_tfs.txt). Among these, genes co‐detectable at both the translatome and transcriptome levels in any tissue were designated as “regulators,” while the remaining genes were classified as target genes. GRNs were constructed based on the expression matrix using GENIE3 (version 3.20) software, which employs a random forest algorithm. Three types of regulatory networks were generated: MmGRNs (transcriptome–transcriptome GRNs), TtGRNs (translatome‐to‐translatome GRNs), and TmGRNs (transcriptome‐to‐translatome GRNs). Each network retained the top one million edges to define high‐confidence regulatory relationships between regulators and target genes [5]. Subsequently, a high‐confidence integrated network, referred to as the MF GRNs, was constructed by merging the edges from the MmGRNs, TtGRNs, and TmGRNs. To tailor the regulatory networks to specific biological processes in individual tissues, TFs specifically expressed in muscle and fat tissues were identified. Regulatory relationships for these TFs were extracted from the mmGRNs, TTGRNs, TmGRNs, and the MF GRNs. As a result, four distinct GRNs were constructed for each tissue, with networks specifically associated with key biological processes in muscle and fat tissues. Then, to precisely focus on the hub GRNs underlying porcine meat production traits, translation regulatory variations associated with the 5’UTR regions of target genes related to meat production traits were identified. These variations were used to filter the MF GRNs, leading to the construction of a hub GRN specifically associated with meat production traits in pigs. In our study, the GRNs were visualized using Gephi, with the network's weighted degree and Eigenvector Centrality calculated. In the visualizations, node size represents the weighted degree, while node color is mapped to Eigenvector Centrality.

### 4.16 Identification of SNPs Regulating TE Through Dual‐Luciferase Reporter Assay

To evaluate the effect of candidate SNPs in the 5’UTR region on gene TE, a dual‐luciferase reporter assay was performed. First, the sequences of the different alleles flanking each candidate SNP were extracted, extending 50 bp upstream and downstream of the SNP. If the extension exceeded the 5’UTR region, only the 5’UTR sequence was retained. The allele‐specific sequences of all candidate SNPs were synthesized by GeneCreate Biological Engineering Co., Ltd (Wuhan, China), and the T7 promoter and each candidate sequence were cloned upstream of the luciferase reporter gene in the pGL3 plasmid. The Firefly luciferase was used as the reporter gene, and Renilla luciferase served as the internal control. PCR amplification of the plasmid generated in vitro transcription DNA templates, which were then transcribed to yield the reporter mRNA containing the target SNP. For cell transfection, Lipofectamine MessengerMAX (Invitrogen, LMRNA008) was employed. Fat tissue‐related SNPs were transfected into porcine primary subcutaneous preadipocytes cells, and muscle tissue‐related SNPs were transfected into porcine primary skeletal muscle cells. After 24 h, cells were harvested, and luciferase activity was measured using a dual‐luciferase detection system (Promega, Glomax20/20) with the Dual‐Glo Luciferase kit (Promega, E1910). The Firefly/Renilla relative fluorescence ratio was calculated. Differences in TE between alleles were analyzed by *t*‐test, with statistical significance defined as *p* < 0.05.

### 4.17 Functional Validation of Target Genes

Target gene‐specific siRNAs and negative controls (NC) were custom‐synthesized by Ribo Biological Co., Ltd (Table S12). For muscle tissue‐related MF GRN targets, the method followed our previously published protocol [6]. C2C12 myoblasts were transfected with the siRNAs using jetPRIME reagent (Polyplus,101000027) when the cells reached approximately 80% confluency. After 8 h, differentiation medium was added to induce myogenic differentiation. A second transfection was performed on day 3 of differentiation, continuing for an additional 3 days. Myogenic differentiation was assessed by immunofluorescence and quantitative PCR (qPCR).

### 4.18 Analysis of Potential Mechanisms of Functional Variants

To evaluate whether 5’UTR functional variants affect gene TE by altering RBP binding, we downloaded all Position Weight Matrices (PWMs) from the AURA database [127], which includes the catalog of inferred sequence binding preferences of RNA‐binding proteins (CIS‐BP‐RNA) [128], as well as from the RNA‐binding protein database (RBPDB) [129]. We then used the MEME Suite's FIMO tool to scan the reference and alternate SNPs within the 5’UTR sequences and compare whether the different alleles resulted in distinct RBP binding patterns (Match *p*‐value < 0.0001). To investigate the impact of SNP variants on RNA structure, we utilized RNAfold software to calculate the minimal free energy (MFE, △G) for both the reference and alternate 5’UTR sequences. The free energy difference between the reference and alternate alleles was used to assess the potential effects of the variant on RNA secondary structure (△△G =|△Galt −△Gref|). Additionally, we employed 3dRNA (v2.0) [130], which combines RNA sequence and RNA secondary structure information, to visualize the 3D conformation of the RNA.

## Author Contributions

C.W., L.F., Y.Z., and Y.L. designed the study. Y.Z., C.C., S.Q., Y.Q., Y.B., H.L., and Z.C. performed the experiments. All bioinformatics analyses were carried out by C.W., with assistance from X.X. and R.C. The manuscript was written and revised by C.W. and Y.L., with Y.Z. providing samples and contributing to the manuscript revision. All authors have read and approved the final manuscript.

## Funding

This work was supported by the National Natural Science Foundation of China (32372858 to Y.W.L.), the Basic Research Center, Innovation Program of the Chinese Academy of Agricultural Sciences (CAAS‐BRC‐LP‐2025‐01 to Y.W.L.), the National Key Research and Development Program of China (2021YFF1000600 to Y.W.L.), the Agricultural Science and Technology Innovation Program of the Chinese Academy of Agricultural Sciences (CAAS‐ZDRW202406 to Y.W.L.), the National Natural Science Foundation of China Young Scientists Fund (32502880 to C.W.), and the Shenzhen Science and Technology Program (Grant No. KJZD20230923115003006 to Y.W.L.).

## Conflicts of Interest

The authors declare no conflicts of interest.

## Ethics Statement

All experimental procedures were approved by the Ethics Committee of Agricultural Genomics Institute at Shenzhen (AGIS‐ER‐2024‐003).

## Supporting information




**Supporting File 1**: advs74200‐sup‐0001‐SuppMat.docx.

**Supporting File 2**: advs74200‐sup‐0002‐TableS1‐TableS12.zip.

## Data Availability

The raw sequence data from RNA‐seq, Ribo‐seq, and WGS generated in this study have been deposited in the Genome Sequence Archive at the BIG Data Center (http://bigd.big.ac.cn/) under the accession code PRJCA039199. The multi‐omics gene standardized expression matrix and GRN information have been submitted to Zenodo (https://doi.org/10.5281/zenodo.15620994).
