# Supplementary material for: Multi‐Tissue Omics Analysis Uncovers Translational Regulation Underlying Complex Traits in Pigs
Source: Adv Sci (Weinh). 2026 Feb 3;13(20):e74200. doi: 10.1002/advs.74200 (PMC13067857; doi:10.1002/advs.74200)
Supplement: Supplementary file 1 — Supporting File 1: advs74200‐sup‐0001‐SuppMat.docx. [file ADVS-13-e74200-s001.docx]

**Supplementary Figures:**


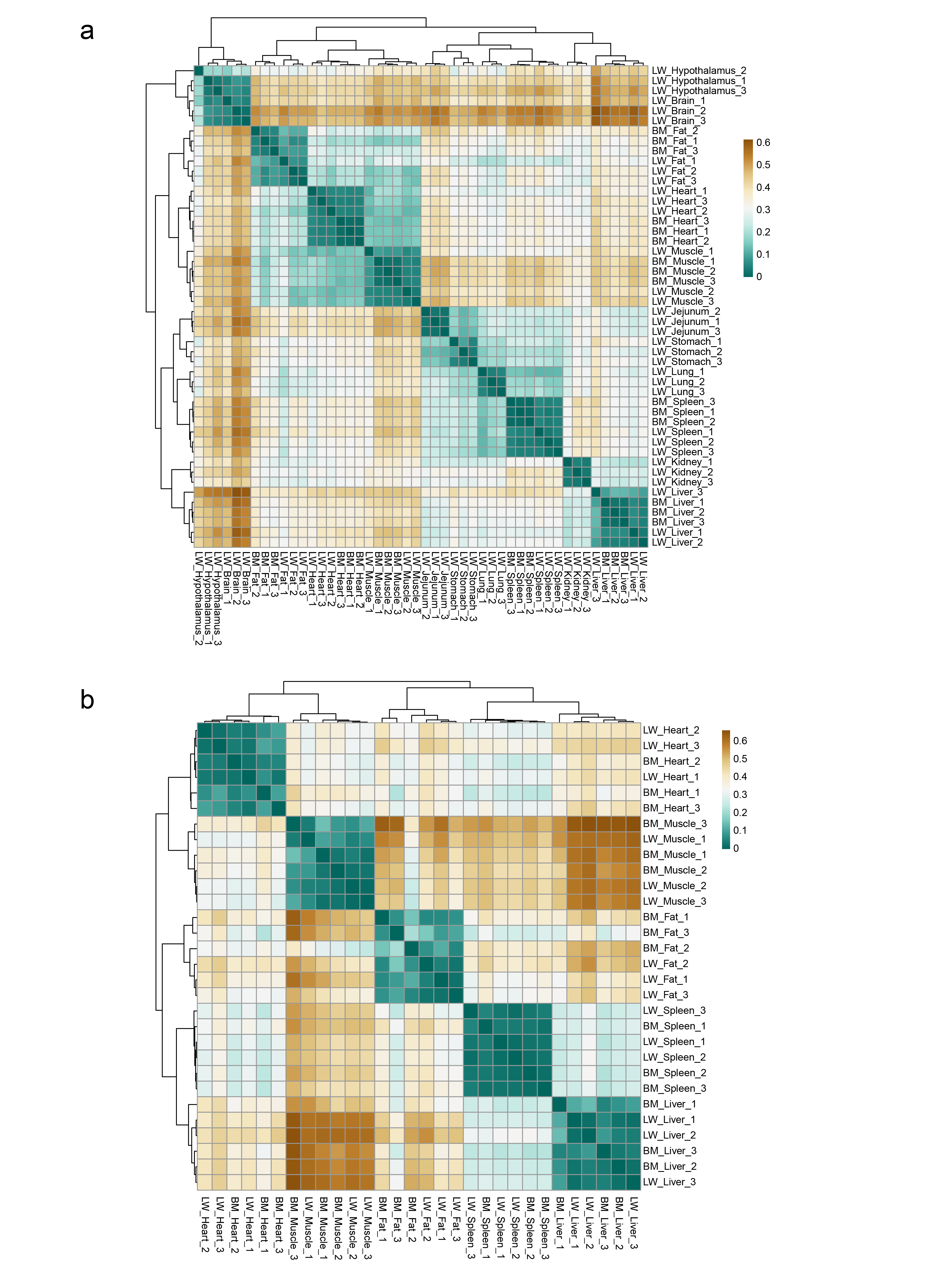


**Figure S1. Euclidean distance-based clustering of samples for RNA-seq and proteomics data.**

**a** Clustering of RNA-seq samples based on Euclidean distances calculated from gene expression profiles. **b** Clustering of proteomics samples based on Euclidean distances calculated from protein abundance data.


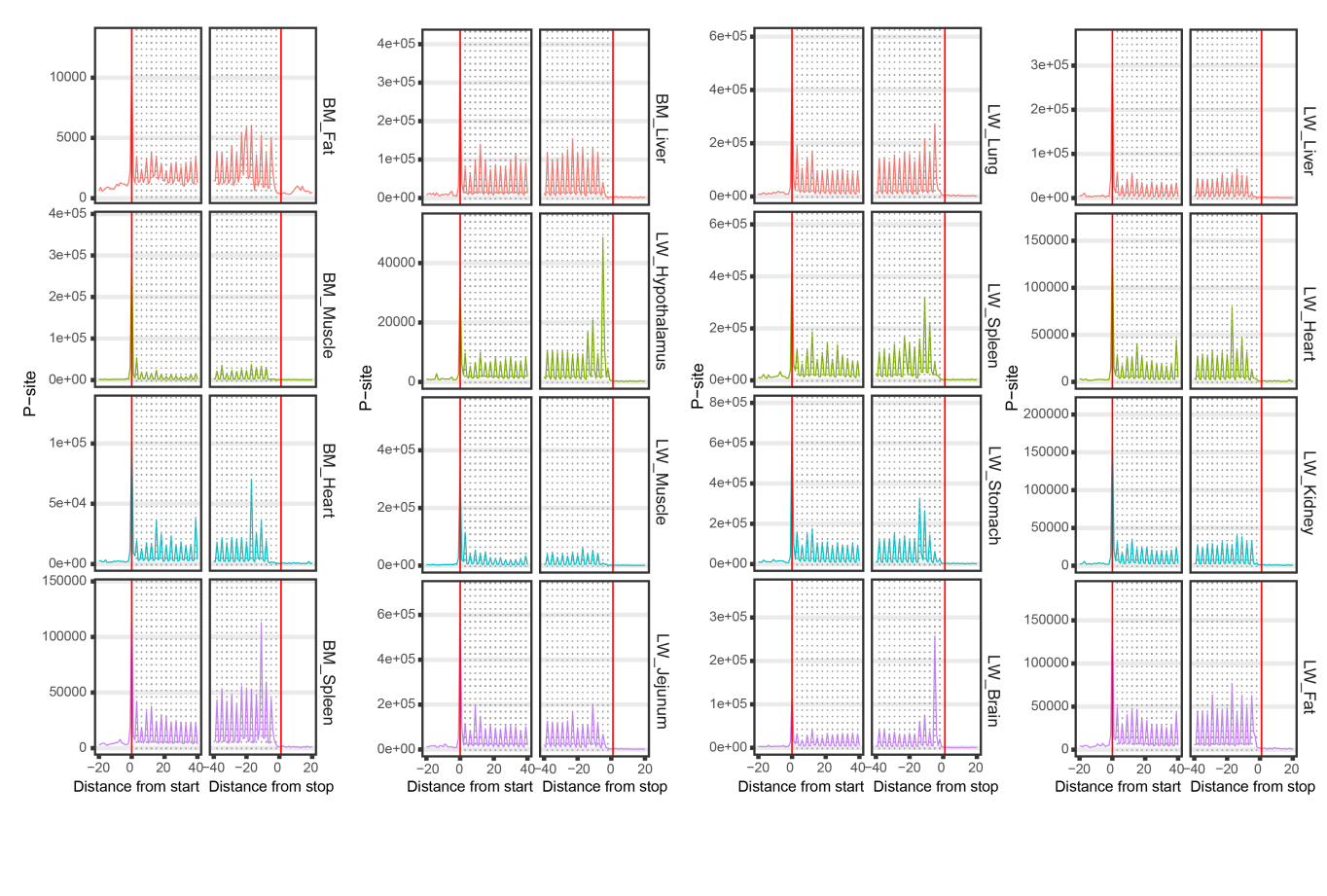


**Figure S2. Distribution of optimally mapped reads along the CDS, with each read represented by its P-site position based on fragment length**


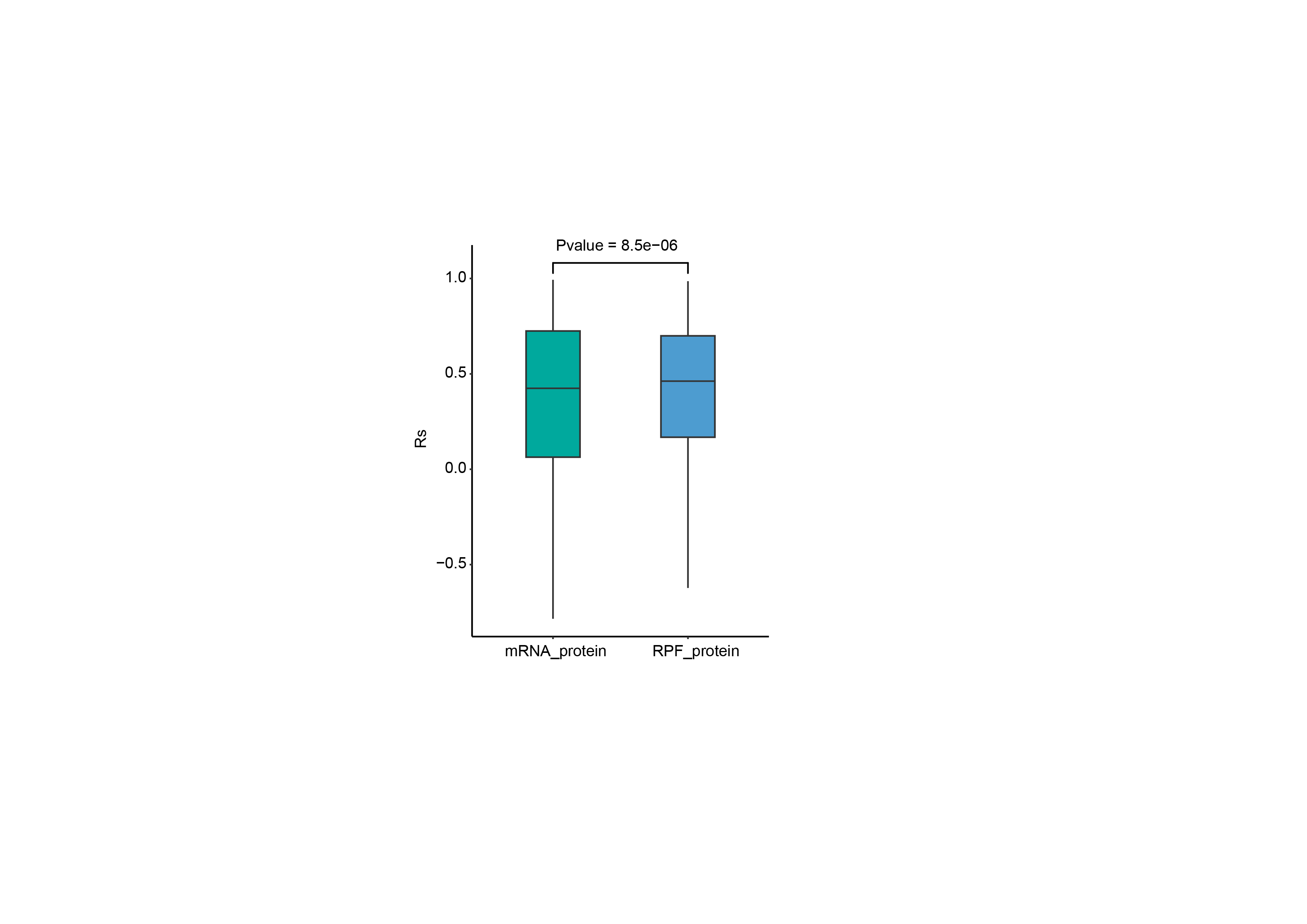


**Figure S3. Comparison of pearson correlation coefficients (r) between inter-sample mRNA vs. protein and inter-sample RPF vs. protein.** Inter-group significance was assessed using the Wilcoxon rank-sum test.


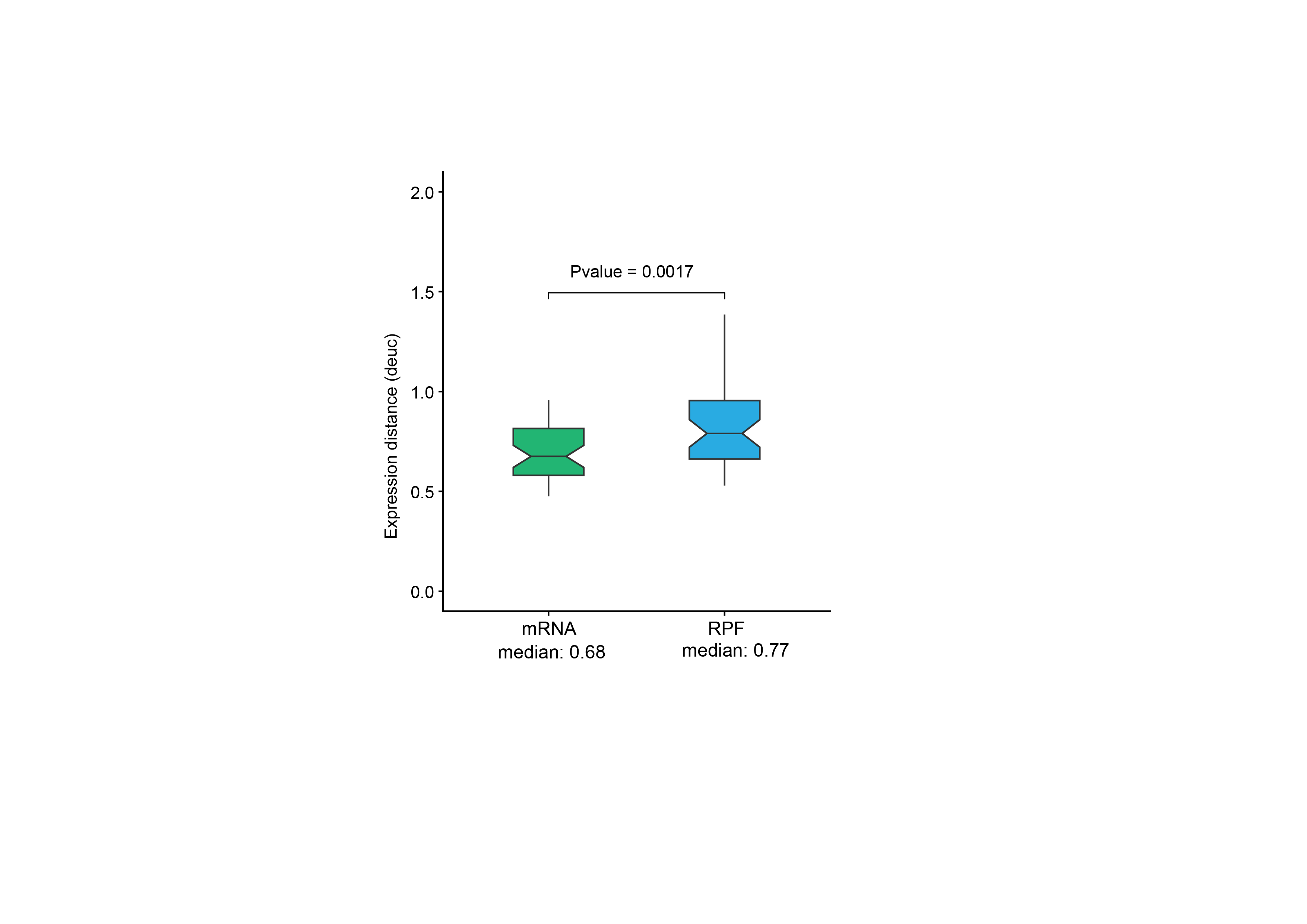


**Figure S4. Divergence of gene mRNA and RPF expression across breeds within the same tissue type.** Inter-group significance was evaluated using the Wilcoxon rank-sum test.

**
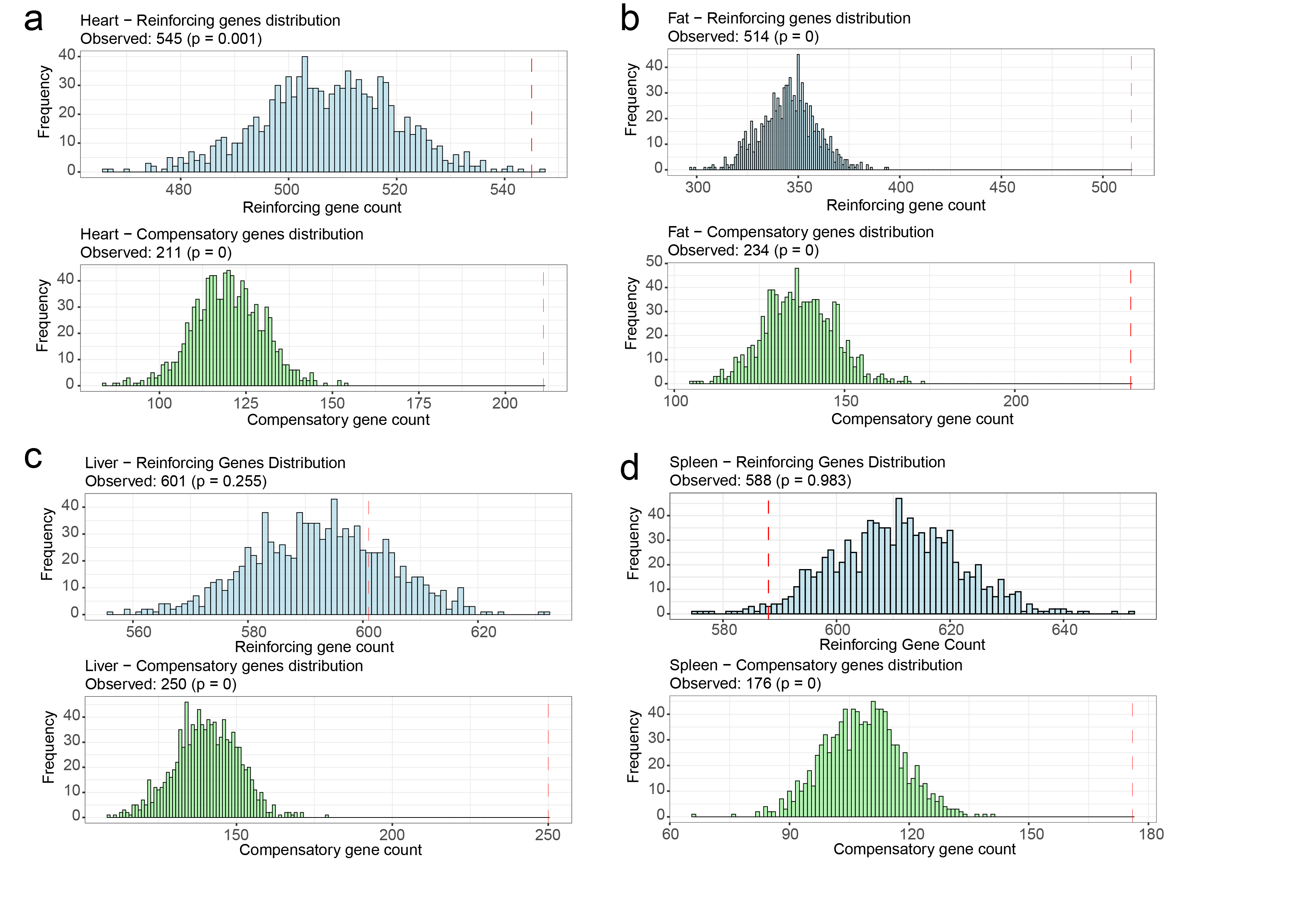
**

**Figure S5. Enrichment of reinforcing and compensatory genes across tissues.**

a–d, Frequency distributions of the numbers of reinforcing and compensatory genes in heart (a), fat (b), liver (c), and spleen (d) based on 1,000 randomized datasets. Red dashed lines indicate counts from the empirical data.

**
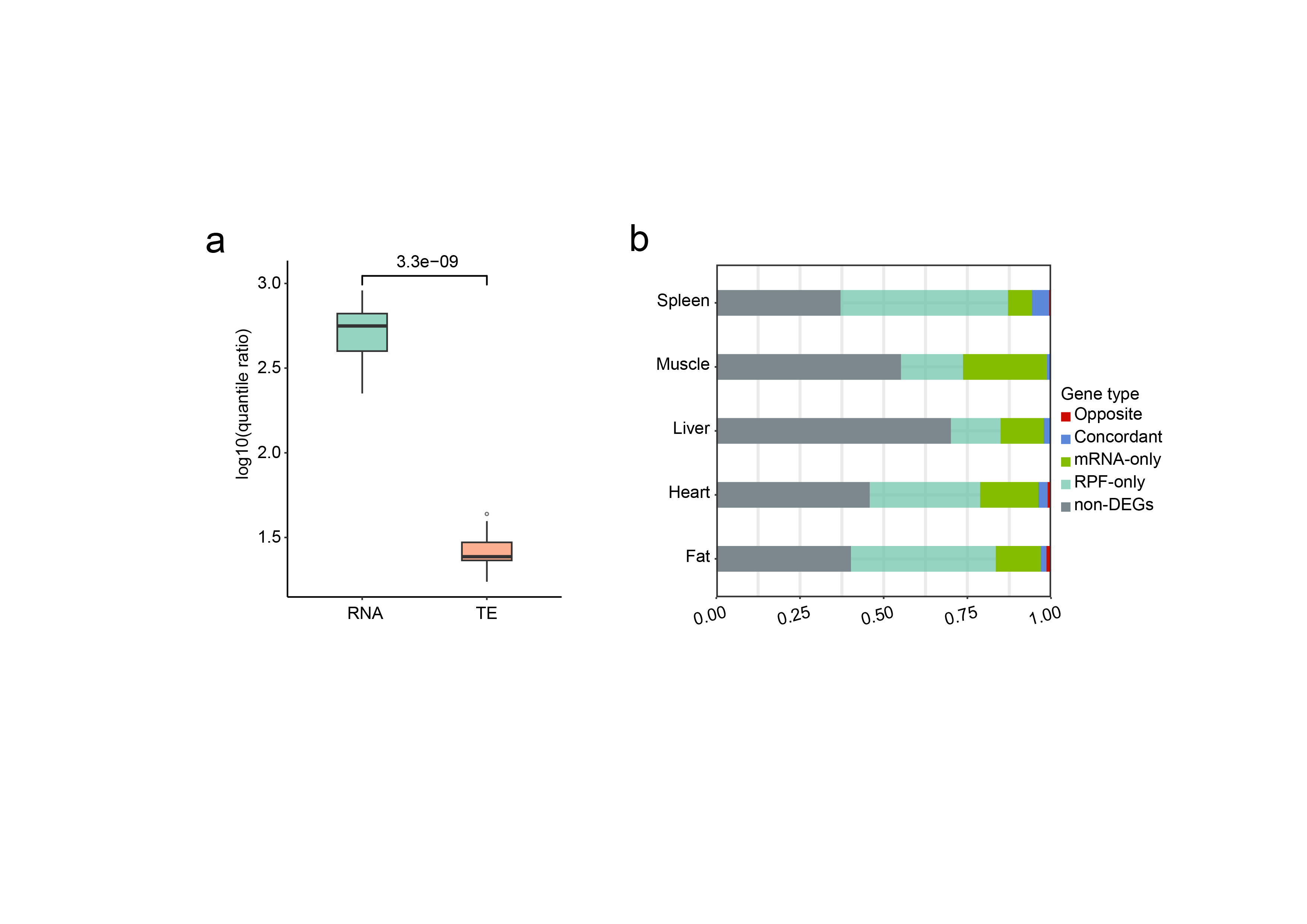
**

**Figure S6. Characterization of gene TE.** Comparison of TE and mRNA expression distribution ranges across tissues, defined as the ratio of the 97.5th to the 2.5th percentile. P-values were calculated using the Wilcoxon rank-sum test.


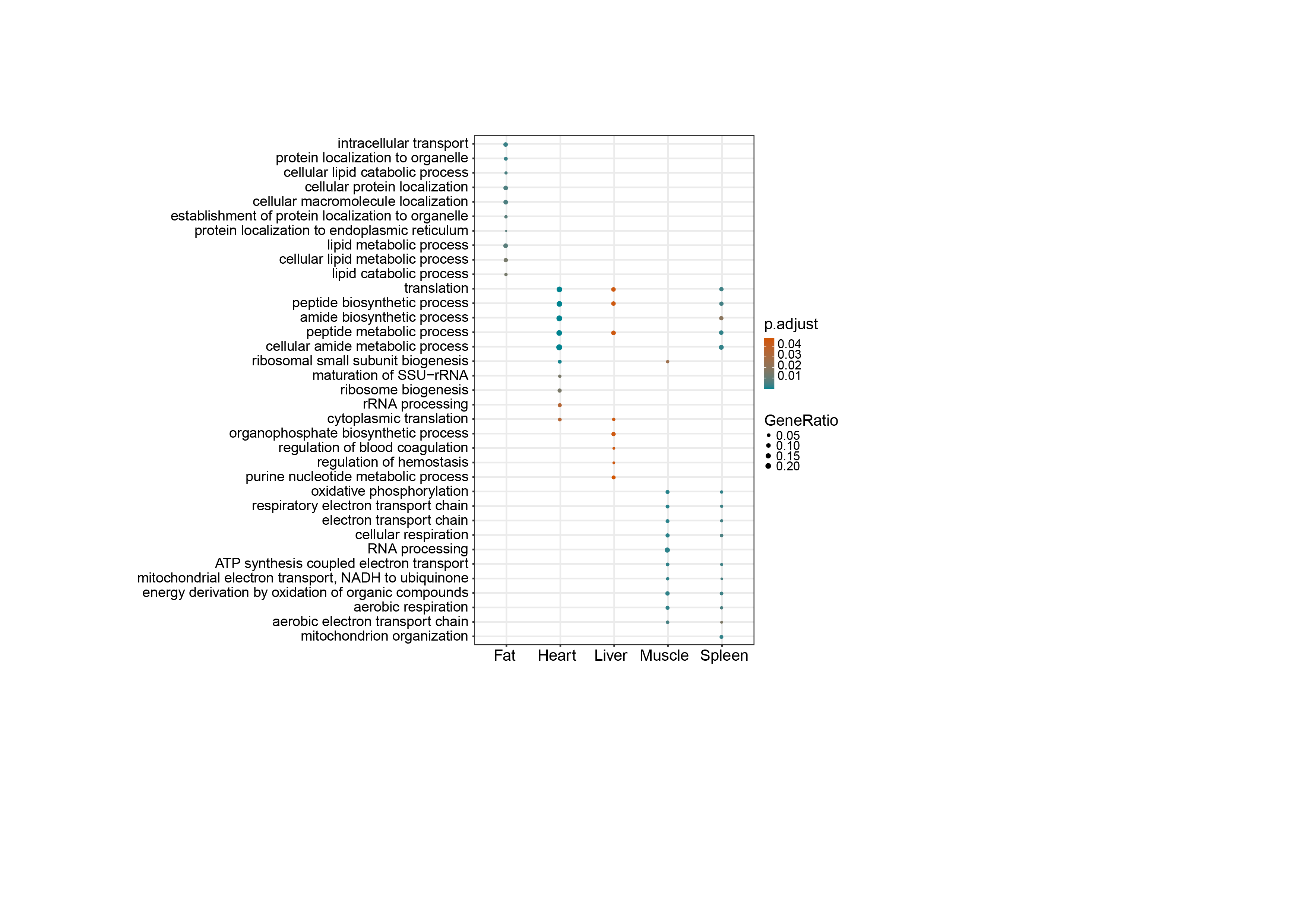


**Figure S7. GO enrichment analysis of genes with differential TE across breeds in five tissues, visualizing the top 10 enriched GO terms for each tissue.**


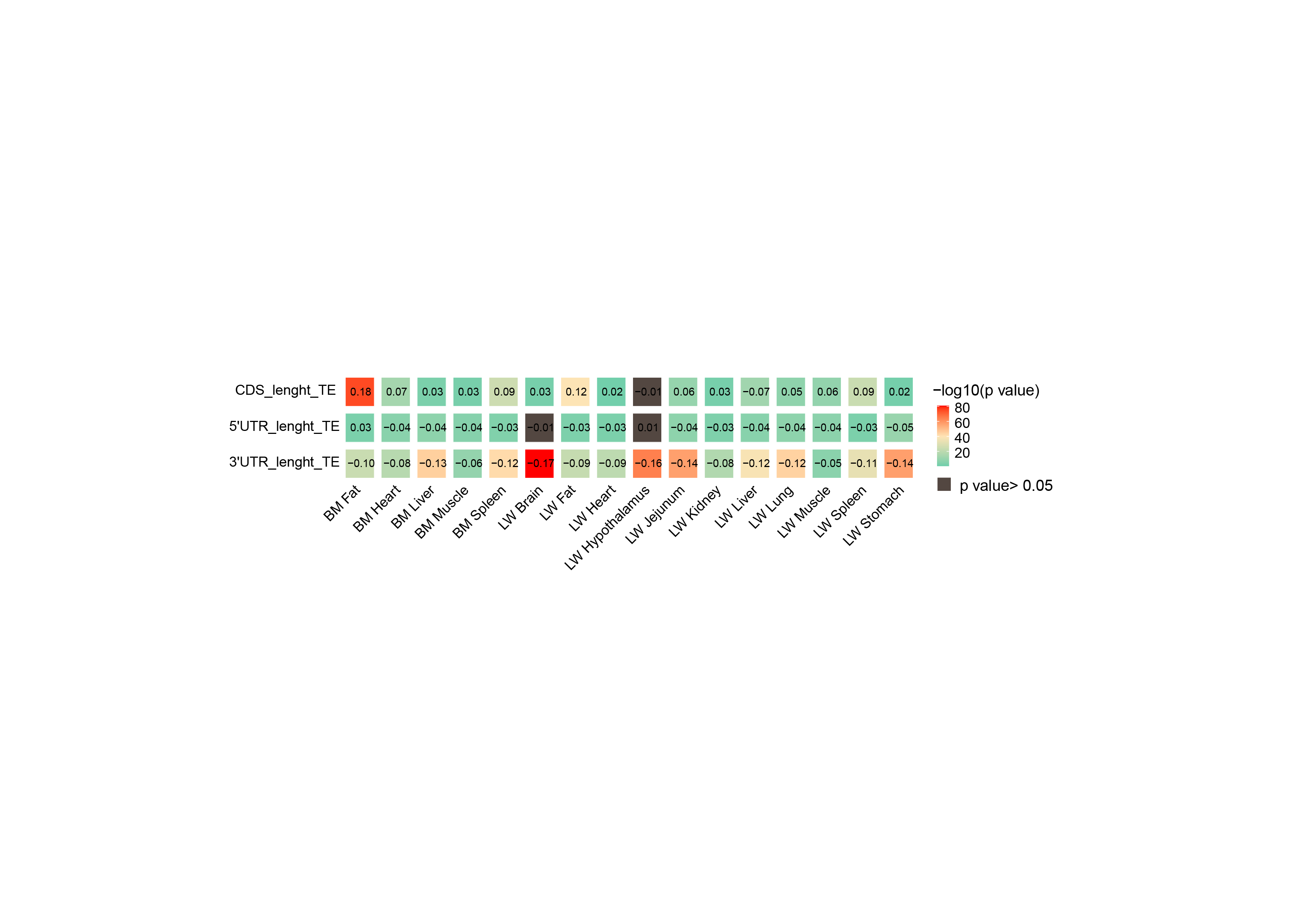


**Figure S8. Correlation between sequence lengths of different gene regions and gene translation efficiency**


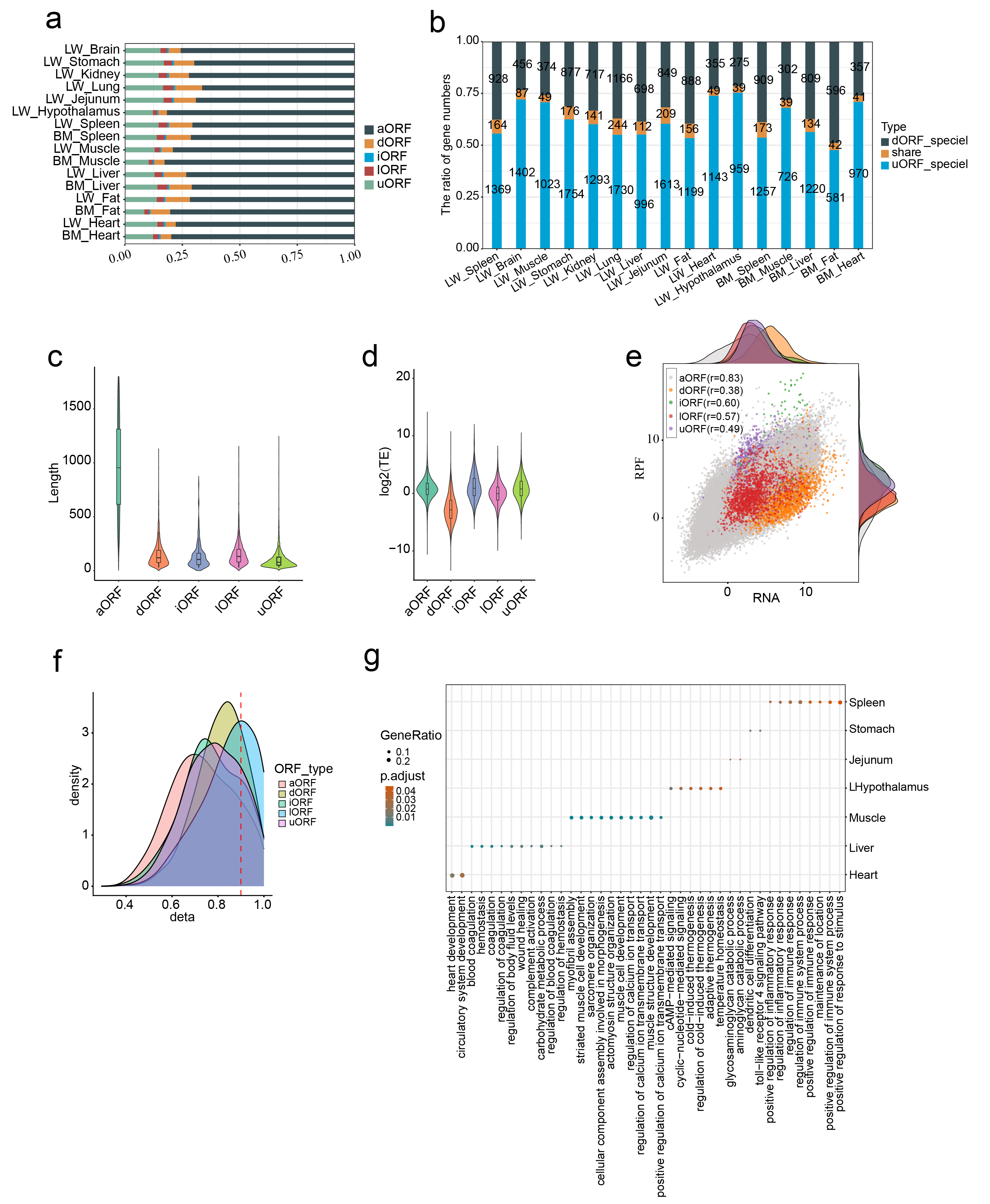


**Figure S9. Identification and features of active translation ORFs.**

**a** Proportion of different types of active ORFs detected in specific tissues of different breeds. **b** Number and proportion of genes in each tissue containing both active translation uORFs and dORFs, only active translation uORFs, or only active translation dORFs. **c** Comparison of the lengths of different types of active ORFs. **d** Distribution of TE across different types of actively translated ORFs. **e** Scatter plot depicting the correlation between RNA and mRNA signals for different ORF types, with the Pearson correlation coefficient (r) determined through correlation analysis. **f** Tissue-specific TE expression indices for different types of ORFs across tissues. **g** GO enrichment analysis of genes containing tissue-specific TE ORFs.


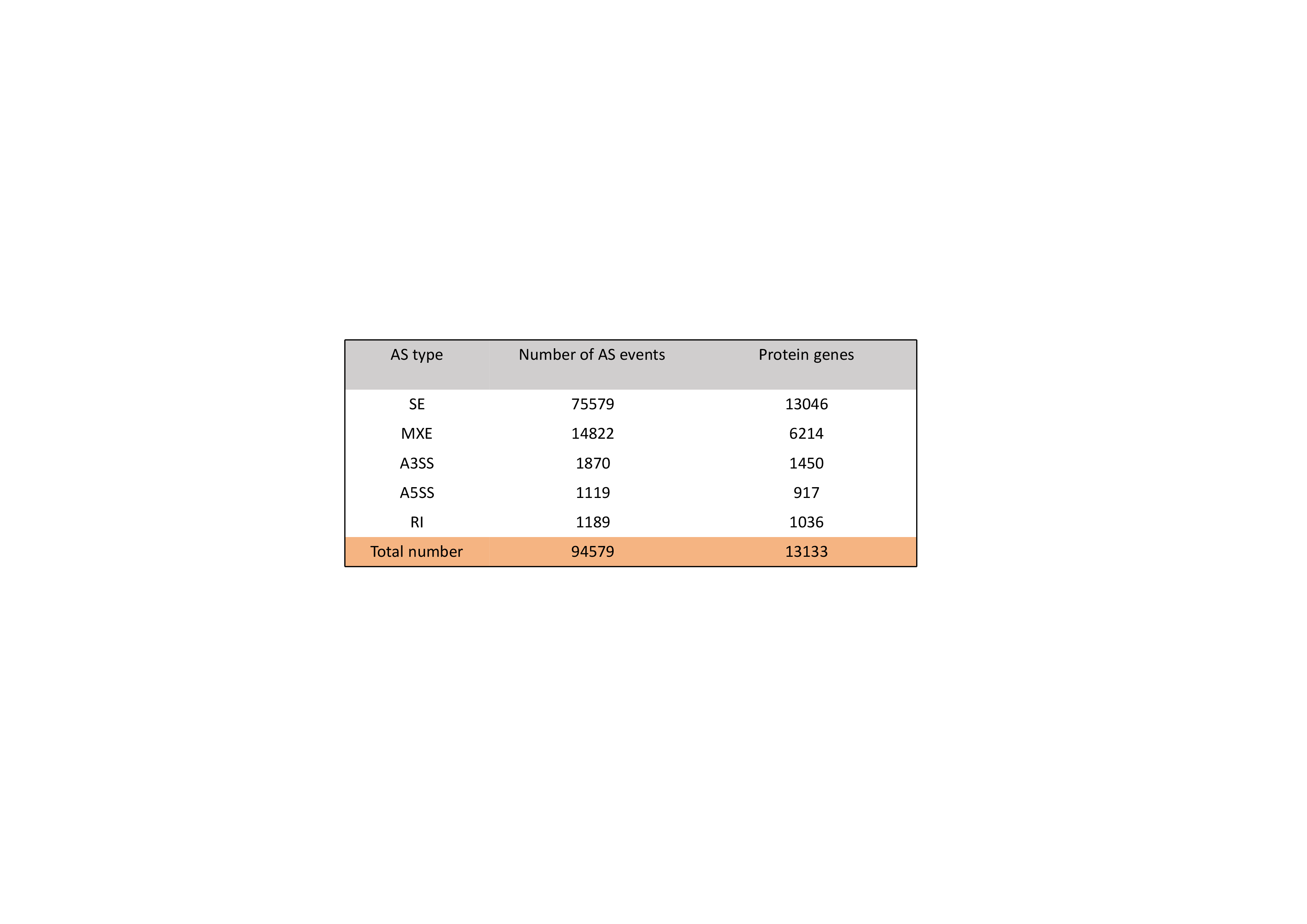


**Figure S10. Total number of each type of alternative splicing event and the total number of protein-coding genes involved in each type, detected across all tissues.**


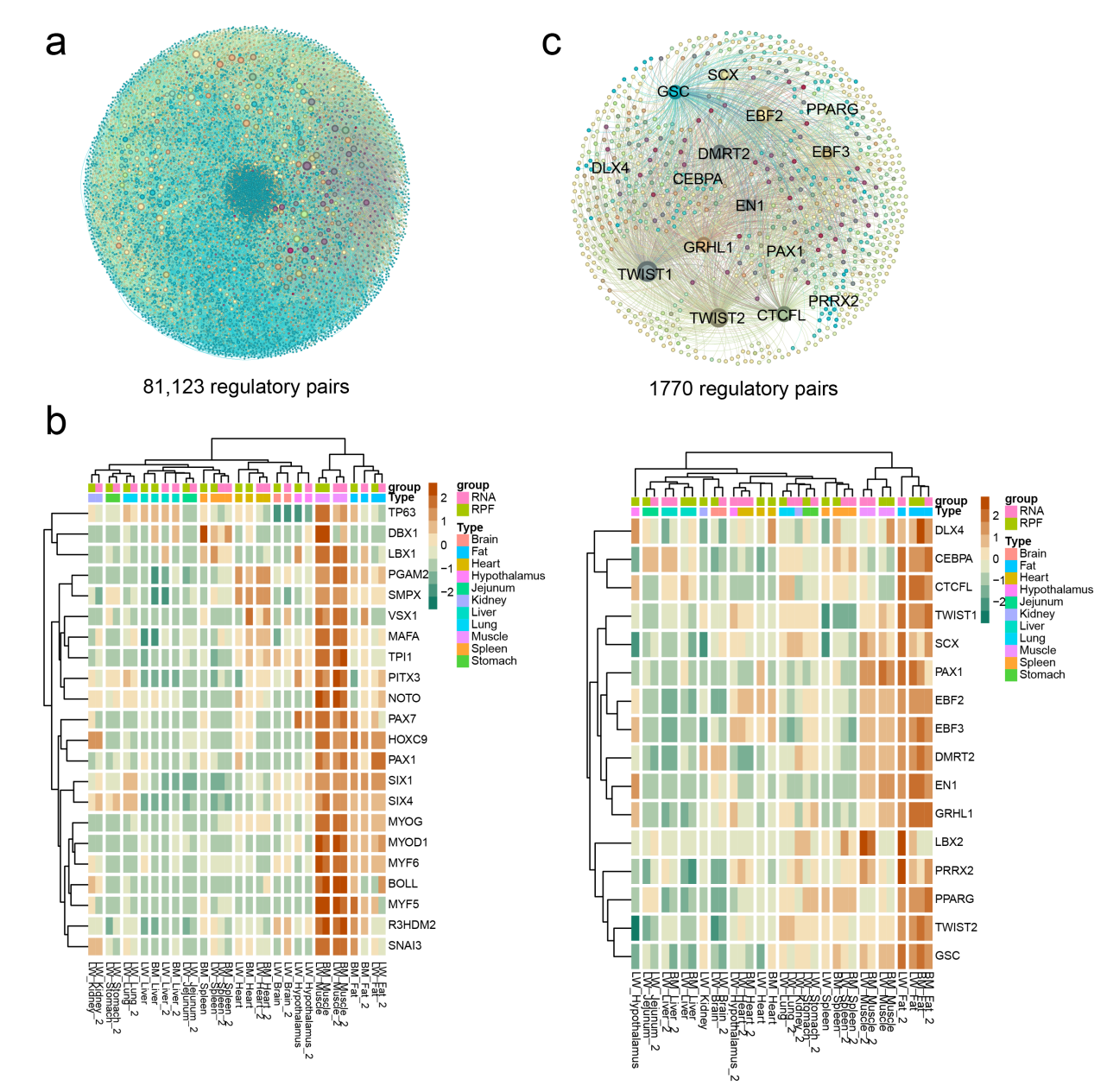


**Figure S11. Visualization of regulatory information for different types of regulatory networks.**

**a** Visualization of the MF GRNs, encompassing a total of 81,123 transcription factor-gene regulatory pairs. **c** Visualization of the core gene regulatory network associated with adipose tissue, selected from the MF GRNs, involving 1,770 transcription factor-gene regulatory pairs. **d** Left: Heatmap showing the dynamic expression of muscle-specific transcription factors across different tissues. Right: Heatmap displaying the dynamic expression of adipose-specific transcription factors across different tissues.

**
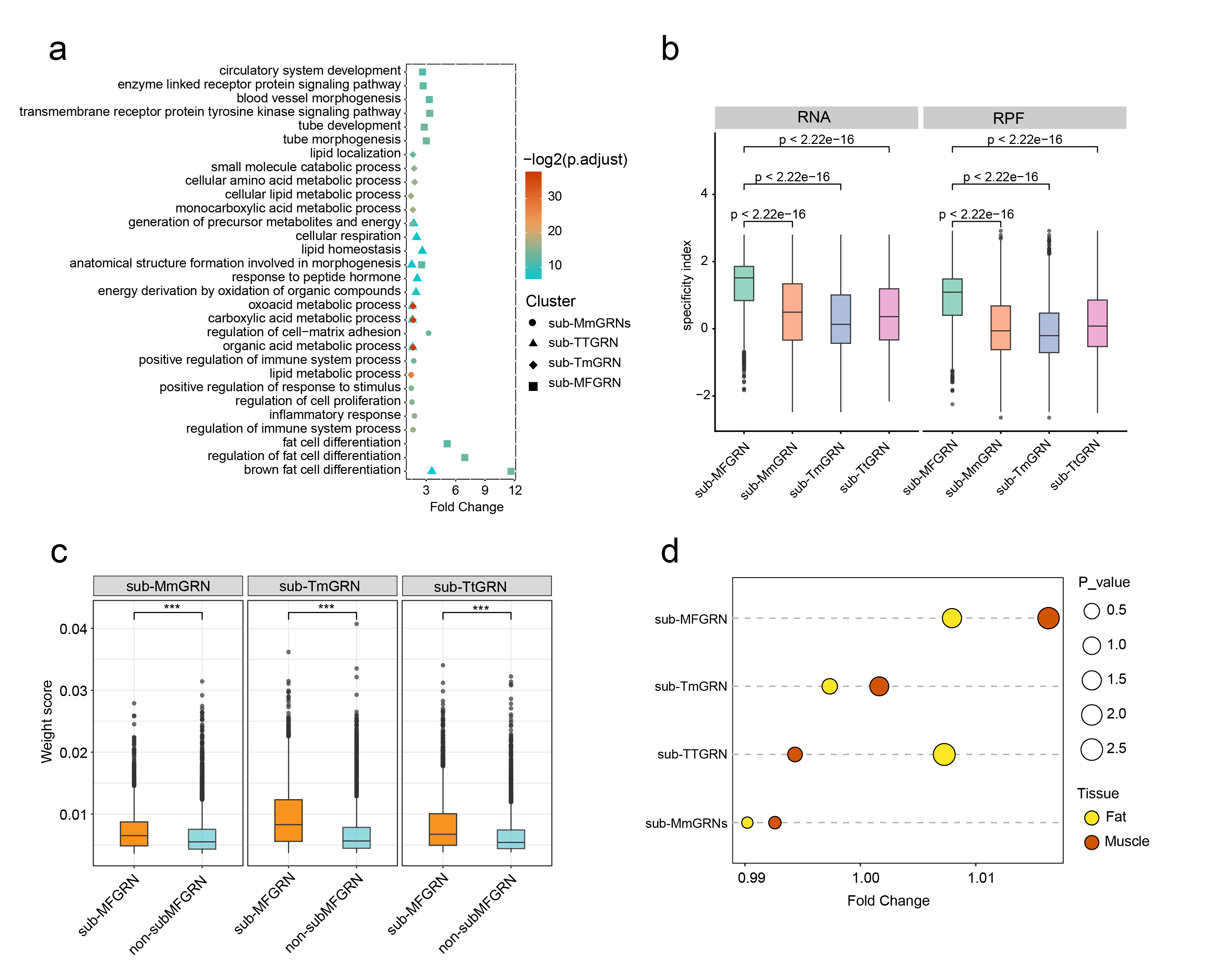
**

**Figure S12. Biological regulatory functions and feature analysis of GRNs.**

**a** Bubble plot showing the GO enrichment analysis results of target genes in the adipose tissue-related sub-GRNs. **b** Comparison of fat tissue-specific expression indices for target genes in different types of sub-GRNs within fat tissue, at both RNA and RPF levels. **c** Comparison of weight scores between target genes in the sub-MF GRN and target genes in non-sub-MF GRNs across various GRNs. **d** Comparison of the enrichment of target genes in muscle and adipose tissue-related sub-GRNs in relation to GWAS signals for pig meat and carcass traits.


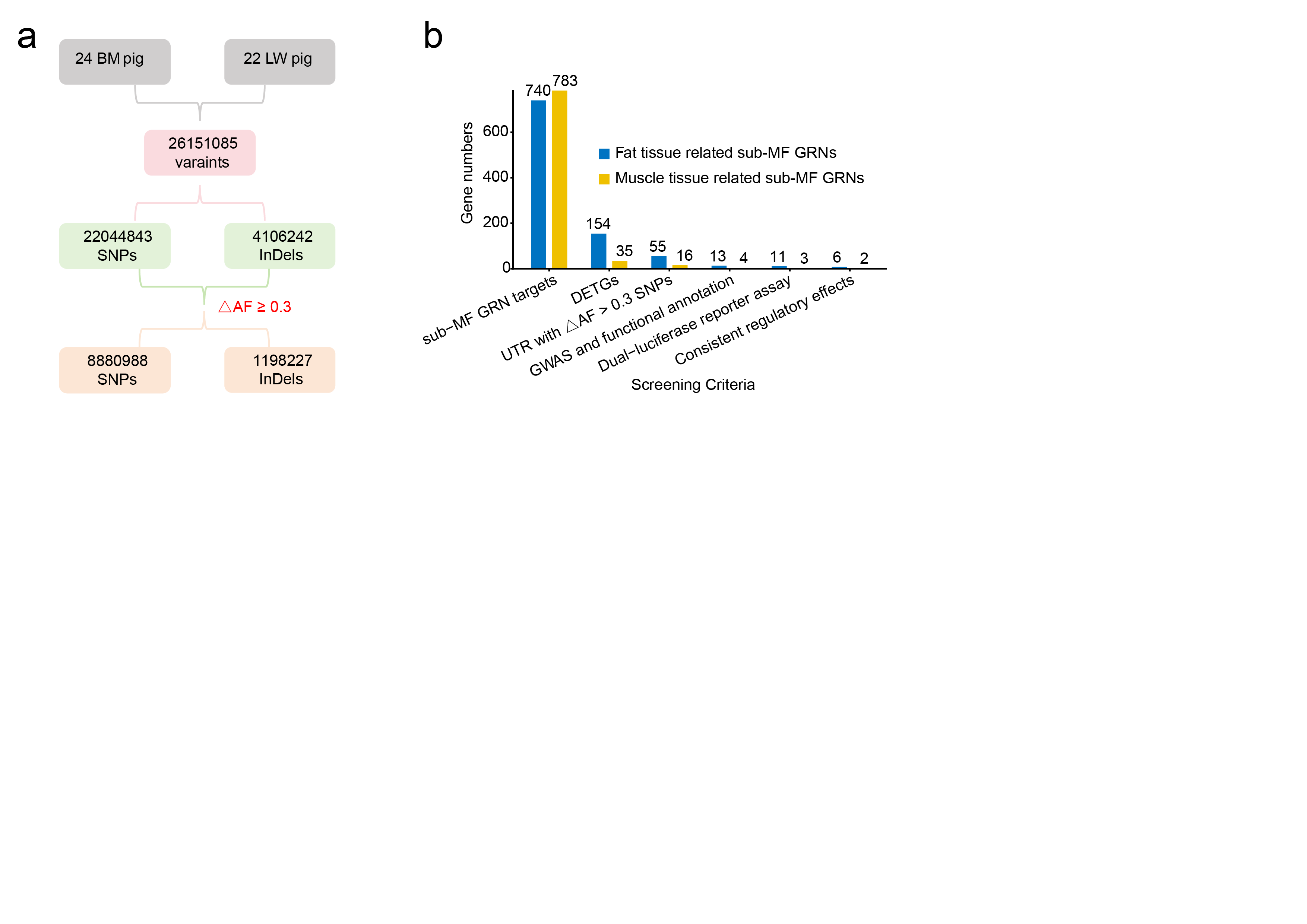


**Figure S13. Biological regulatory functions and feature analysis of GRNs.**

**a** Genetic variant screening based on allele frequency differentiation between breeds.

**b** Functional gene screening system for trait regulation within translational regulatory networks.

**
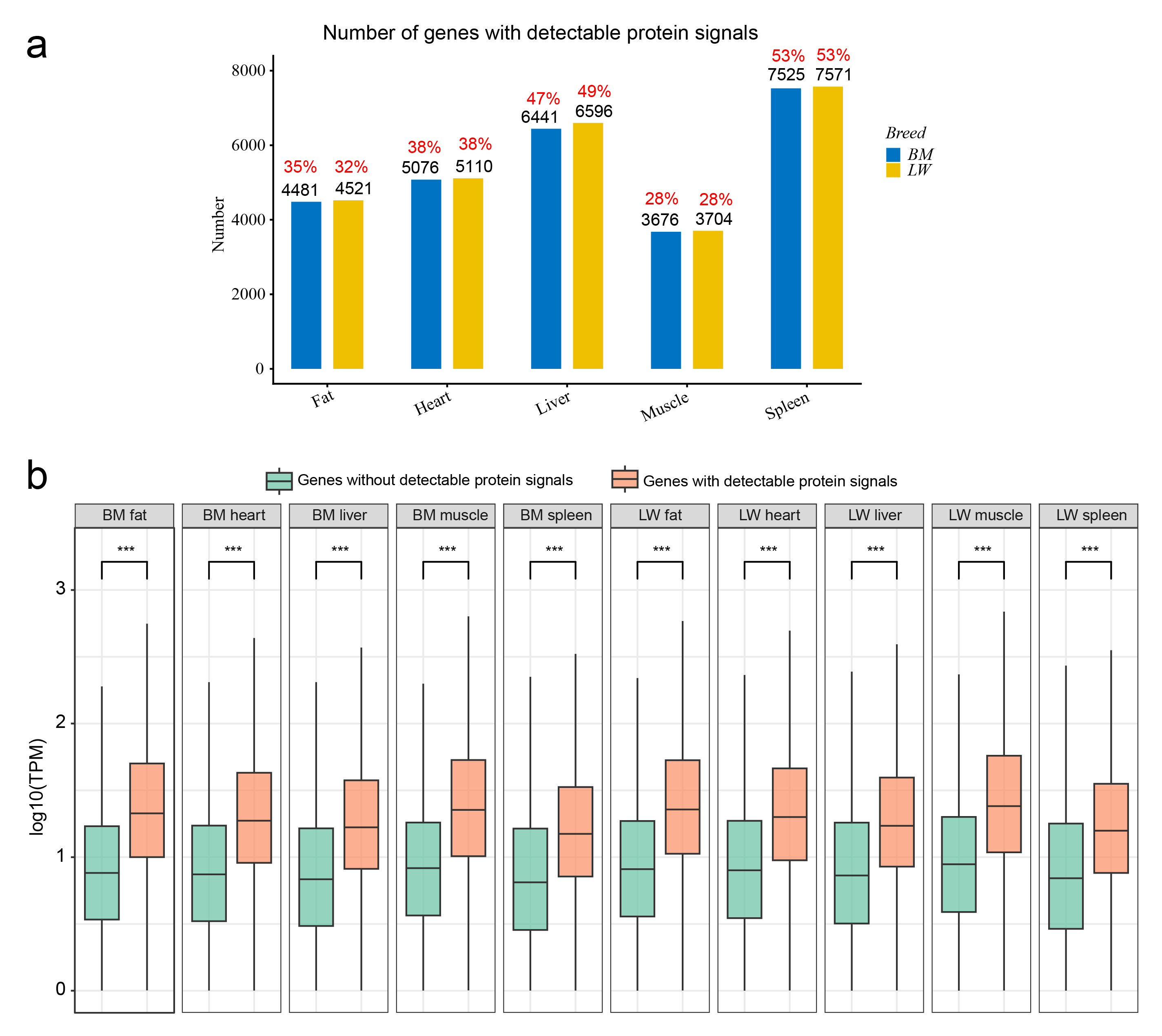
**

**Figure S14. Protein signaling detection sensitivity is limited by gene transcriptional activity.**

**a** Number and proportion of genes with detectable protein signals among genes with detectable transcriptional signals in each tissue. **b** Comparison of transcriptional activity between genes with detectable protein signals and those without detectable protein signals among genes with detectable transcriptional signals in each tissue. *** indicates P-value < 0.01, with P-values calculated using the Wilcoxon rank-sum test.

**Figure S15.
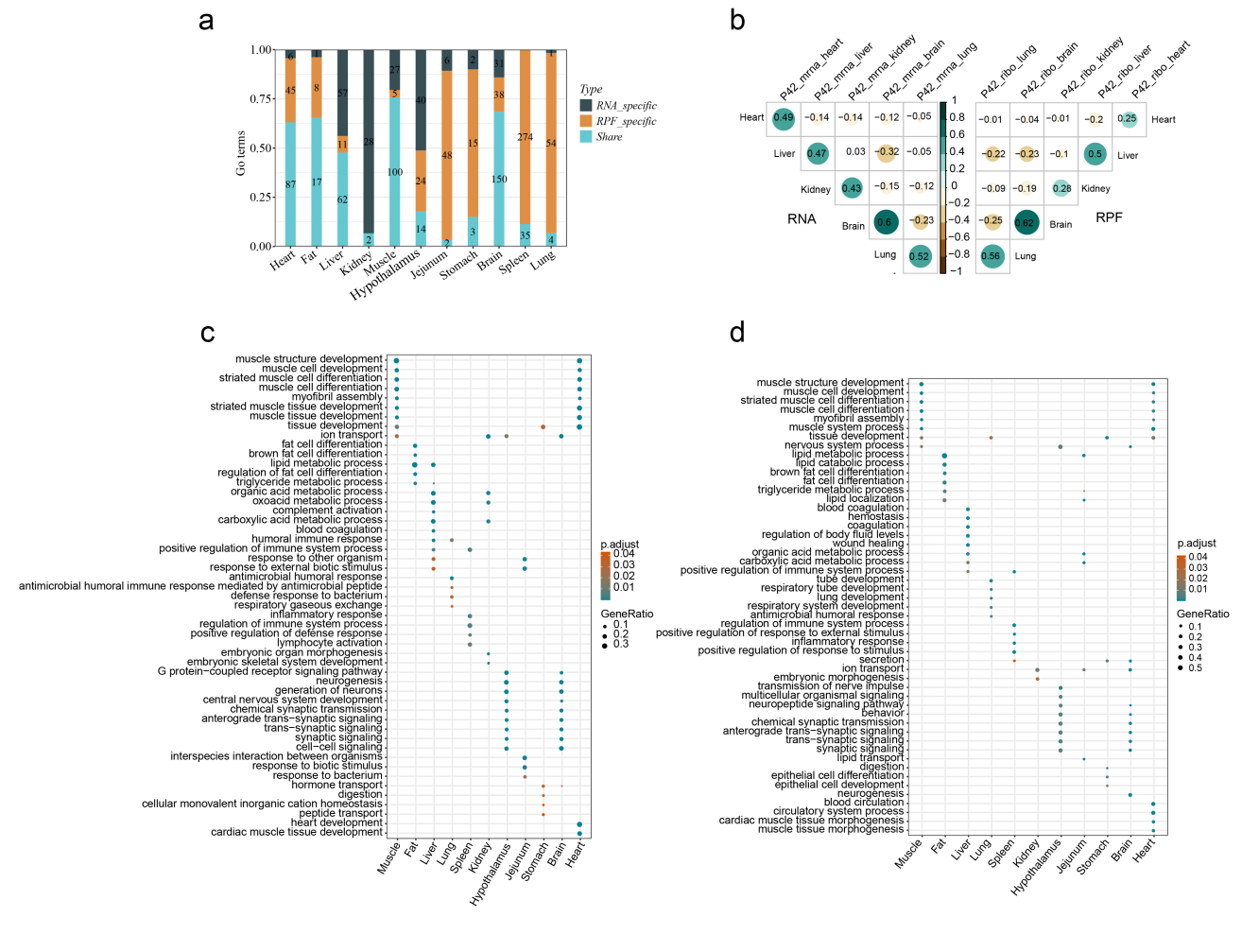
Integrated analysis of tissue-specific gene expression across mRNA and RPF levels.**

**a** Proportional bar plot showing the overlap of significantly enriched GO terms between tissue-specific mRNA and RPF gene sets RNA_specific represents pathways significantly enriched in mRNA tissue-specific genes, RPF_specific represents pathways enriched in RPF-specific genes, and Share represents shared GO terms enriched in both mRNA and RPF tissue-specific gene sets. **b** Cross-species conservation of tissue specificity for mRNA and RPF expression (pig vs. mouse). **c** GO enrichment analysis of tissue-specific mRNA abundance genes. Point size indicates the degree of enrichment, and color reflects statistical significance. **d** GO enrichment analysis of tissue-specific RPF abundance genes. Point size indicates the degree of enrichment, and color reflects statistical significance.

**
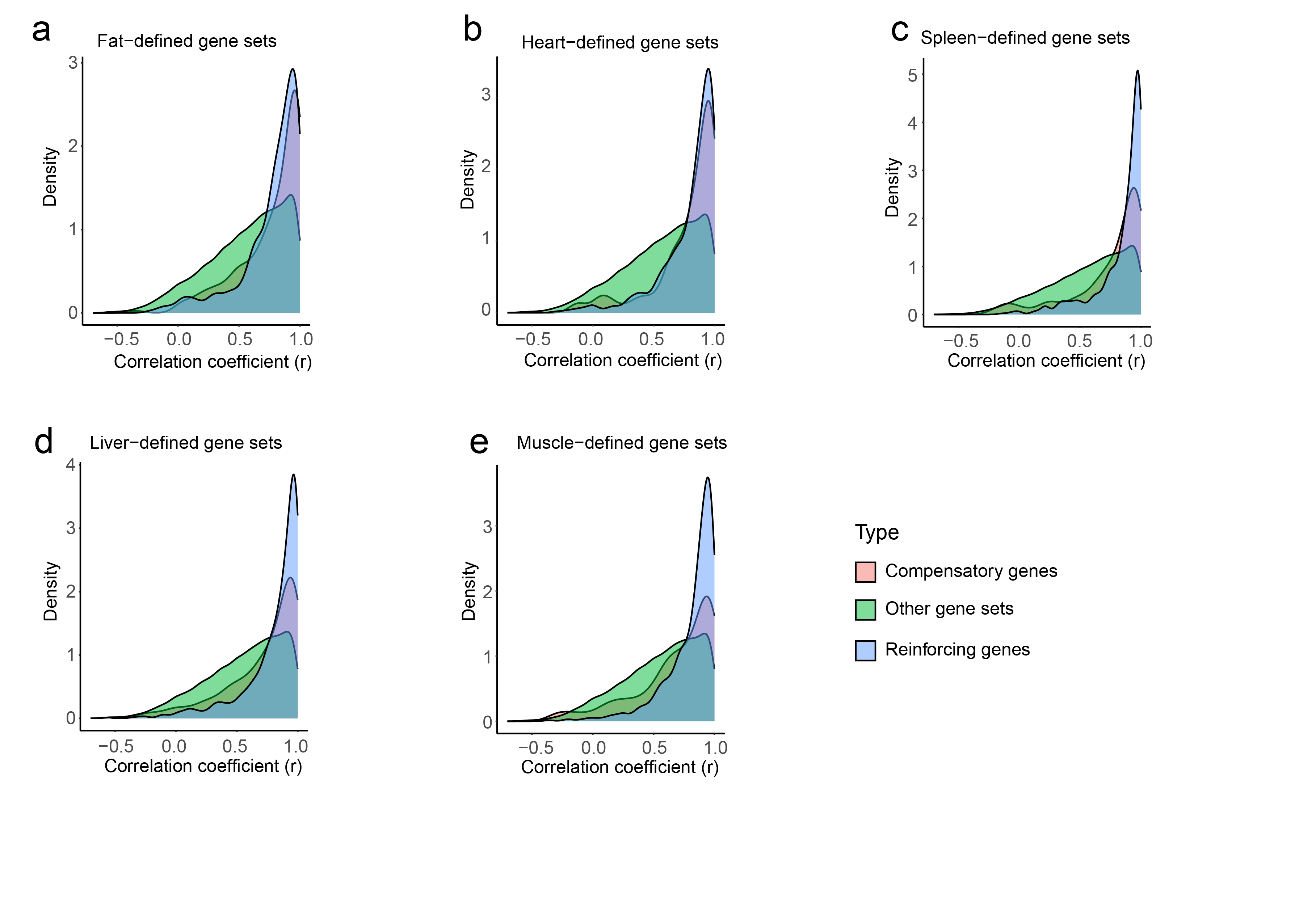
**

**Figure S16. Density distributions of cross-tissue correlations for mRNA and RPF levels in each gene category for diffirent tissues.**


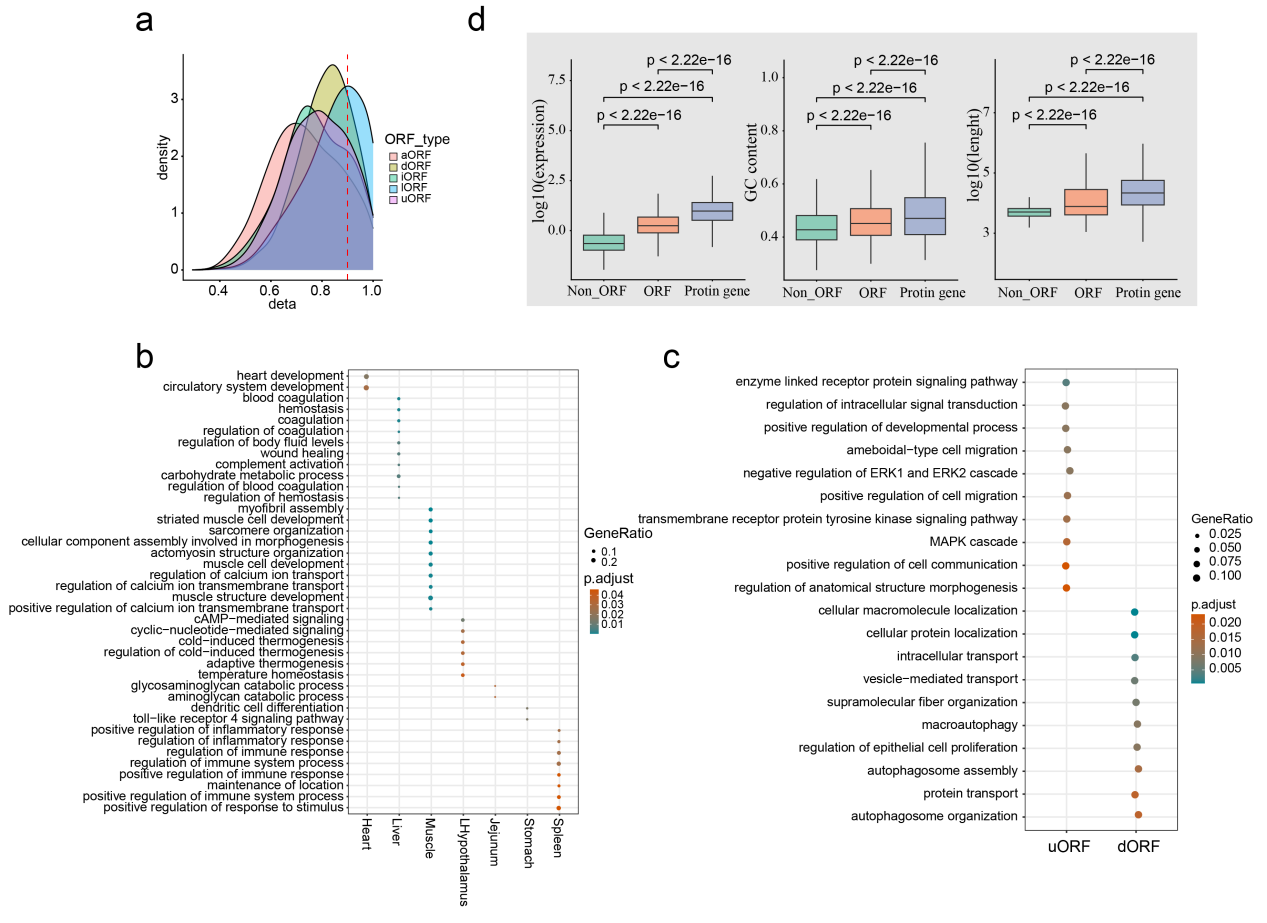


**Figure S17. Functional regulation analysis of actively translated ORFs**

**a** Tissue-specific TE expression indices for different types of ORFs across tissues. **b** Comparison of RNA expression levels among lncRNAs containing actively translated lORFs, lncRNAs without actively translated ORFs, and protein-coding genes (Wilcoxon test, P < 0.01). **c** GO enrichment analysis of genes containing tissue-specific TE ORFs. **d** GO enrichment analyses showing that genes containing actively translated uORFs and dORFs are involved in distinct biological functions.


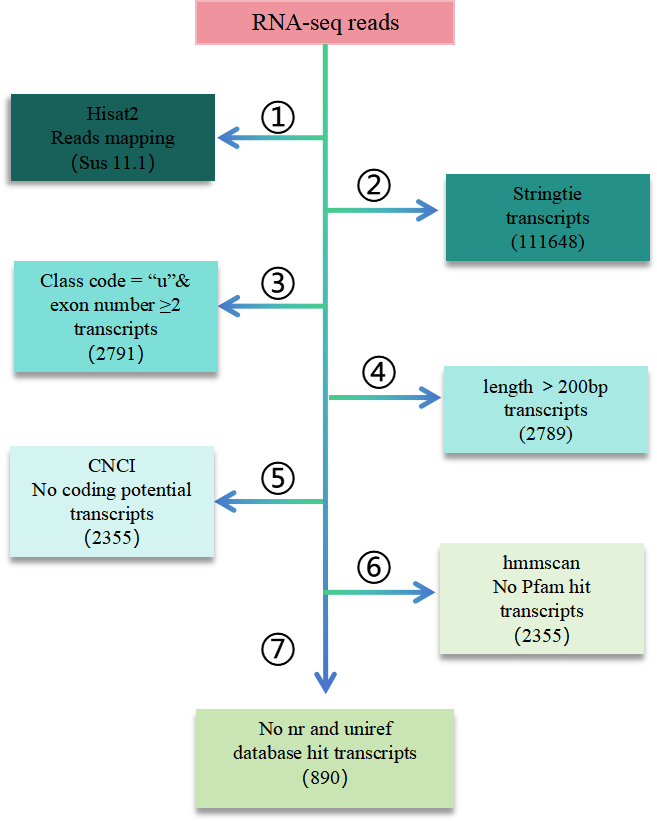


**Figure S18. Diagram of the novel lncRNA identification pipeline.**
